# PARP1 expression drives the synergistic antitumor activity of trabectedin and PARP1 inhibitors in sarcoma preclinical models

**DOI:** 10.1186/s12943-017-0652-5

**Published:** 2017-04-28

**Authors:** Ymera Pignochino, Federica Capozzi, Lorenzo D’Ambrosio, Carmine Dell’Aglio, Marco Basiricò, Marta Canta, Annalisa Lorenzato, Francesca Vignolo Lutati, Sandra Aliberti, Erica Palesandro, Paola Boccone, Danilo Galizia, Sara Miano, Giulia Chiabotto, Lucia Napione, Loretta Gammaitoni, Dario Sangiolo, Maria Serena Benassi, Barbara Pasini, Giovanna Chiorino, Massimo Aglietta, Giovanni Grignani

**Affiliations:** 1 Sarcoma Unit, Medical Oncology, Candiolo Cancer Institute - FPO, IRCCS, Candiolo, Torino, Italy; 2 Department of Oncology, University of Torino Medical School, Candiolo, Torino, Italy; 3 Pathology Unit, Candiolo Cancer Institute - FPO, IRCCS, Candiolo, Torino, Italy; 4 Medical Oncology, Candiolo Cancer Institute – FPO, IRCCS, Candiolo, Torino, Italy; 5 Department of Genetics, Biology and Biochemistry, University of Torino, Torino, Italy; 6 Laboratory of Vascular Oncology, Candiolo Cancer Institute – FPO, IRCCS, Candiolo, Torino, Italy; 7 Current address: Department of Applied Science and Technology, Politecnico di Torino, Torino, Italy; 8 Laboratory of Vascular Oncology, Candiolo Cancer Institute - FPO, IRCCS, Candiolo, Torino, Italy; 9 Experimental Oncology Laboratory, IRCCS Istituto Ortopedico Rizzoli, Bologna, Italy; 10 Cancer Genomics Lab, Fondazione Edo ed Elvo Tempia, Biella, Italy

**Keywords:** Predictive biomarkers, PARP1 inhibitors, DNA-damaging agents, Trabectedin, Olaparib, Bone and soft tissue sarcomas, Drug synergism

## Abstract

**Background:**

Enhancing the antitumor activity of the DNA-damaging drugs is an attractive strategy to improve current treatment options. Trabectedin is an isoquinoline alkylating agent with a peculiar mechanism of action. It binds to minor groove of DNA inducing single- and double-strand-breaks. These kinds of damage lead to the activation of PARP1, a first-line enzyme in DNA-damage response pathways. We hypothesized that PARP1 targeting could perpetuate trabectedin-induced DNA damage in tumor cells leading finally to cell death.

**Methods:**

We investigated trabectedin and PARP1 inhibitor synergism in several tumor histotypes both in vitro and in vivo (subcutaneous and orthotopic tumor xenografts in mice). We searched for key determinants of drug synergism by comparative genomic hybridization (aCGH) and gene expression profiling (GEP) and validated their functional role.

**Results:**

Trabectedin activated PARP1 enzyme and the combination with PARP1 inhibitors potentiated DNA damage, cell cycle arrest at G2/M checkpoint and apoptosis, if compared to single agents. Olaparib was the most active PARP1 inhibitor to combine with trabectedin and we confirmed the antitumor and antimetastatic activity of trabectedin/olaparib combination in mice models. However, we observed different degree of trabectedin/olaparib synergism among different cell lines. Namely, in DMR leiomyosarcoma models the combination was significantly more active than single agents, while in SJSA-1 osteosarcoma models no further advantage was obtained if compared to trabectedin alone. aCGH and GEP revealed that key components of DNA-repair pathways were involved in trabectedin/olaparib synergism. In particular, PARP1 expression dictated the degree of the synergism. Indeed, trabectedin/olaparib synergism was increased after PARP1 overexpression and reduced after PARP1 silencing.

**Conclusions:**

PARP1 inhibition potentiated trabectedin activity in a PARP1-dependent manner and PARP1 expression in tumor cells might be a useful predictive biomarker that deserves clinical evaluation.

**Electronic supplementary material:**

The online version of this article (doi:10.1186/s12943-017-0652-5) contains supplementary material, which is available to authorized users.

## Background

An attractive strategy to improve current treatment options is to inflict cytotoxic DNA damage with chemotherapy, and then impede DNA repair by molecular targeting. Poly-ADP-ribosyl-transferase-1 (PARP1) is a key sensor of DNA damage and initiates recruitment of the DNA-repair machinery to the site of damage [1–3]. The development of PARP1 inhibitors has drawn closer the goal of combining these compounds with current therapies [2, 4–9]. Unfortunately, despite several preclinical data confirming an increased antitumor activity by combining PARP1 inhibitors with either chemotherapy or radiotherapy [10–16], dose escalation in phase 1 combination studies has been greatly hampered by the observed hematologic toxicities. These adverse events limited the possibility to exploit these combinations into clinical practice [4, 5, 9, 11, 17–27]. As a consequence, PARP1 inhibitors are today registered as monotherapy in cancers bearing DNA-repair deficiencies [28–34] and the strong rational to combine selected cytotoxics (especially alkylators) with PARP1 inhibitors has to face the risk of myelotoxicity.

Among chemotherapeutics, trabectedin has some peculiarities that point out this drug as an ideal candidate to be combined with PARP1 inhibitors: its favorable safety profile on the hematologic side and its unique mechanism of action [35, 36]. In particular, trabectedin traps enzymes belonging to the transcription-coupled nucleotide excision repair (TC-NER) system that in the attempt to remove trabectedin adducts, generates DNA single- and double-strand breaks. Understandably, trabectedin displays a greater clinical benefit in BRCA1/2-deficient tumors than in proficient ones [37, 38]. These characteristics prompted us to explore the combination of trabectedin and PARP1 inhibitors taking advantage of a large set of bone and soft tissue sarcoma (BSTS) cell lines. Despite a common mesenchymal origin, BSTS are characterized by a great degree of heterogeneity and, indeed, trabectedin displays a spectrum of activity in various sarcoma histotypes [39–41]. This heterogeneity allowed us to test cells with different intrinsic sensitivity to trabectedin, in order to explore if trabectedin could efficiently activate PARP1, and if PARP1-specific inhibition could be exploited sufficiently to cause irreversible DNA damage, and eventually tumor cell death. The observed results were subsequently validated in other tumor types and by combining PARP1 inhibitors with other cytotoxics characterized by different mechanisms of action.

## Methods

### Cell line characterization, cell viability and western blot

Cell lines characteristics are depicted in Additional file 1: Table S1 and S2. Short tandem repeat (STR) profile was checked and the genomic status of DNA-repair key components (BRCA1, BRCA2, ATM, CHEK2, PTEN) was analyzed by Multiplex ligation-dependent probe amplification (MLPA) and Denaturing high performance liquid chromatography (DHPLC, Wave 3500HT DNA Fragment Analysis System, Transgenomic Inc.) followed by direct sequencing (ABI PRISM3100 DNA Sequencer, Applied Biosystem, Forster City, CA). PARP1, RAD51, and BRCA1 copy number variations was confirmed by real-time polymerase chain reaction (PCR) on genomic DNA (TaqMan Assay, ABI PRISM 7900HT System, Applied Biosystem).

In order to evaluate the proliferation rate of each cell line, cells were seeded at a density of 2 × 10^4^ cells/cm^2^, and the doubling-time (DT) of the harvested cells was calculated during the exponential growth phase (48 h of culture) by using the algorithm provided by http://www.doubling-time.com/compute.php: DT = t x lg_2_/(lgNt - lgN_0_) where N_0_ is the initial concentration of cells, Nt is final concentration of cells and t is the culture time in hours. Cell viability was determined with Cell Titer-Glo (Promega) after 72-h treatment with scalar doses (2–0.125 nM) of trabectedin (PharmaMar), as single agent or in constant combination with olaparib (20–1.25 μM) or veliparib (80–5 μM) (Sequoia research products). These ranges of concentrations were chosen on the results of previous studies testing the sensitivity to each single agent in sarcoma and non-sarcoma cell lines [42–45]. Protein extracts were obtained after 24-h treatment and resolved by western blot using primary antibodies from Cell Signaling Technology except for anti- poly(ADP-ribose) (PAR, Trevigen), phospho-histone H2AX (Ser139) (Millipore).

### Quantitative real-time polymerase chain reaction (PCR), gene expression profiling and Gene Signature enrichment analysis (GSEA)

2.5 × 10^6^ cells were plated in 150 mm diameter and grown for 24 h in complete medium. Cells were then treated for additional 24 h with trabectedin (0.125 nM) and olaparib (1.25 μM) as single agent and in combination. In each condition, 500.000 cells were fixed in 70% ethanol for cell cycle analysis and the remaining fraction was lysed with Qiazol reagent (Qiagen) for RNA extraction by means of RNeasy Kit (Qiagen) following manufacturer’s instructions. Gene expression profiling was performed with Human HT-12 v4.0 Expression BeadChip Kit (Illumina) Real-time PCR was performed by TaqMan Gene Expression Assay using ABI PRISM 7900HT System (Applied Biosystem). Fluorescence data were automatically converted into C_T_ (cycle threshold) values. To export data, the threshold was 0.20. Raw data were analyzed by Microsoft Office Excel. Additional file 2: Figure S1 outlined the design of the expression profile project analyzed by GSEA (http://www.broadinstitute.org/gsea/msigdb/index.jsp) [46]. To highlight gene patterns associated and involved with trabectedin and olaparib synergism, we designed a specific expression profile project comparing cell lines displaying high synergism (HS-C: TC-106, 402.91, DMR) and cell lines displaying low/no synergism (LS-C: SJSA-1, HT1080, SW684). First, we compared gene expression profiles of HS-C and LS-C by GSEA to identify gene sets associated with high synergism. Then we selected the cores of the emerging gene sets matching the criterion of false discovery rate (FDR) < 0.05 and we challenged them with GSEA comparing each treated HS-C against untreated. GSEA analysis was performed with default parameters. Probes were collapsed into gene symbols. Gene set size for inclusion was set between 15 and 500, and the genes were permutated 1,000 times. Gene sets that met the FDR < 0.05 were considered significant. The catalog of gene sets was downloaded from Molecular Signature Database (C2, C5, C6, Hallmarks, MSigDB, version 5.0, http://www.broadinstitute.org/gsea/msigdb/index.jsp) for a total of 4965 curated gene sets.

### Array comparative genomic hybridization (aCGH) analysis

Comparative genomic hybridization using aCGH microarrays was carried out using the enzymatic labeling method. Digestion, labeling, hybridization, washing and slide scanning were performed following the manufacturer protocols (Agilent Technologies). Briefly, 750 ng of samples and control DNA in a total volume of 10.1 μL were digested with restriction enzymes and labeled with Cy3 and Cy5. Subsequently, labeled DNA was cleaned up and hybridized using the Agilent-030587 CCMC CGH plus SNP 180 k Microarray platform. Each test DNA sample (Cy5) was hybridized with a reference DNA (Cy3) from Homo sapiens (Agilent). After hybridization for 24 h at 20 rpm and 65 °C, slides were washed following Agilent procedure and scanned with the dual-laser microarray scanner version C (G2505C, Agilent Technologies). Images were analyzed using Feature Extraction software version 10.7 (Agilent Technologies). Raw data were processed using the Agilent Genomic Workbench version 7. Aberrant regions were detected using ADM-2 algorithm with threshold = 6. To avoid false positive calls, the minimum number of consecutive probes for amplifications/deletions was set to 3, together with a minimum average absolute Log Ratio for aberrations > = 0.25.

### Gene ontology (GO) enrichment analysis

Genes mapped in the regions with differential aberrations between HS-C and LS-C were uploaded into DAVID bioinformatics tool for functional enrichment analysis and GO biological processes (level 5). Enrichment p-values less than 0.01 were considered as statistically significant.

### Mice xenograft models

Nonobese diabetic/severe combined immunodeficient (NOD/SCID) mice (Charles River) were injected with: a) 1–2.5 × 10^6^ SJSA-1 subcutaneously (s.c.) into the right flank or 10^5^ SJSA-1 intravenously (i.v.) into the tail vein to originate subcutaneous primary tumors and lung metastases, respectively, or b) 10^6^ DMR s.c. or 4 × 10^5^ DMR orthotopically into the uterine wall by laparotomy. Six mice per group (DMR s.c. model) were randomized to receive: a) 25 or b) 50 mg/kg/day intraperitoneal (i.p) injection of olaparib (5 days/week); c) 0.150 or d) 0.1 or e) 0.05 mg/kg weekly i.v. injection of trabectedin; f) combination of 25 mg/kg/day olaparib and 0.050 mg/kg trabectedin or g) left untreated for 21 days and then sacrificed for histological and molecular assays. SJSA-1 s.c. models were treated with a) 0.05 mg/kg or b) 0.025 mg/kg weekly i.v. injection of trabectedin; c) 25 mg/kg olaparib i.p; d) combination of 25 mg/kg/day olaparib and 0.050 mg/kg trabectedin; e) combination of 25 mg/kg/day olaparib and 0.025 mg/kg trabectedin or g) left untreated for 17 days. DMR orthotopic model was treated with 0.050 mg/kg trabectedin and 25 mg/kg/day olaparib as single agent, in combination, or left untreated. These in vivo experiments were conducted in accordance with the protocol approved by the Institutional Ethics Review Board (IRB) and by the Italian Ministry of Health (Aut. Min. 178/201S-PR). For in vivo imaging, each mouse received an intra-peritoneal dose of 150 mg luciferin/kg body weight, and ventral and dorsal images were obtained by IVIS Lumina II and quantified using Living Image software (PerkinElmer).

### Flow cytometry assays: cell cycle, P-H2AX, and apoptosis analysis

The effects of trabectedin and olaparib on the cell cycle and the expression of the DNA-damage marker P-H2AX were determined by staining DNA content with propidium iodide (PI, Sigma Aldrich) and FITC-conjugated primary antibody anti-P-H2AX (Ser139) (Millipore), respectively, after treatment with trabectedin (0.125 nM) as single agent or in combination with olaparib (1.25 μM) for 24 h.

For apoptosis evaluation, after 96-h treatment APC-labelled Annexin V (eBioscence) and PI (0.5 μg/ml) staining was done. All samples were acquired by Cyan ADP Flow cytometer (Beckman Coulter) and analyzed by the Summit v4.3 (Dako) and FlowJo (Tree Star) softwares.

### Comet assay (Single Cell Gel Electrophoresis assay)

For determination of DNA single- and double-strand breaks, a single-cell gel electrophoresis assay was used (Comet Assay TM, Trevigen) per the manufacturer’s instructions after 48-h treatment with trabectedin (0.125 nM) and olaparib (1.25 μM), as single agents or in combination. Quantization of the DNA in the tails of the comets was performed with Quantity One software (Bio-Rad Laboratories) and ImageJ software version 1.49 (http://rsbweb.nih.gov/ij/index.html).

### Lentiviral vector production

pCCL.sin.cPPT.polyA.CTE.eGFP.minhCMV.hPGK.Luciferase.Wprep or lentiviral plasmid vector PLKO-puro (Sigma-Aldrich), the Precision LentiORF PARP1 (Id:PLOHS_100004266, Thermo Scientific) and packaging vectors pMDLg/pRRE pRSV-REV and pMD2.VSVG were used for lentiviral preparation. Efficiency of transduction was confirmed by western blot analysis and flow cytometry analysis by Cyan ADP Flow cytometer (Beckman Coulter). The luciferase activity was tested by in vitro bioluminescent assay (Caliper Life Sciences, Inc.) and measured by IVIS Lumina II (PerkinElmer).

### Archival tumor samples, immunostaining, and terminal deoxynucleotidyl transferase dUTP nick end labeling (TUNEL) assay

Archival formalin-fixed, paraffin-embedded (FFPE) tumor samples were collected in accordance with an IRB approved protocol (202/2014). Four μm slices were cut and stained with primary antibodies against PARP1, BRCA1 and RAD51 (Abcam) following standard immunohistochemistry procedure. The same antibodies and one antibody against PAR (Calbiochem) were used for immunocytochemistry on cells grown as monolayer in chamber slides. TUNEL assays were used to evaluate the number of apoptotic cells in tumors explanted from mice, using the ApopTag kit (Millipore). Immunostaining on mice xenografts was performed according to standard protocols with primary antibodies from Sigma Aldrich (proliferating cell nuclear antigen, PCNA), Abcam (p-H2AX Serine 139). Nuclei were counterstained with hematoxylin. Immunofluorescence was done with FITC-conjugated antibody against P-H2AX (Millipore). Nuclei were counterstained with DAPI. Fluorescence in situ hybridization (FISH) analysis of PARP1 and centromeres was performed following standard procedures.

Visible images were acquired with a Leica DM1000 microscope equipped with a color 3.1 M Pixel CMOS digital camera. Fluorescent images were acquired with a Leica confocal laser-scanning microscope (TCS SP5 AOBS). For signal quantification, 5 images/sample were acquired by maintaining constant parameters. Image quantification was performed using ImageJ software and Leica Confocal Software.

### Statistical and pharmacological combination analyses

All in vitro experiments were performed at least three times. Differences between treatment groups were analyzed by the two-tailed Student’s *t* test and the two-way ANOVA with post hoc Bonferroni’s correction for multiple tests using GraphPad Prism 5 (GraphPad Software Inc.). The concentration inhibiting 50% of the cell growth (IC50) with its 95% confidence intervals (95% CI), and drug synergism, expressed as combination index (CI) calculated at IC50 with its estimated standard deviation were obtained by using CalcuSyn software (Biosoft). The correlation analyses between ΔCT values (mRNA levels) or protein expression levels and combination indexes were performed by calculating Pearson correlation coefficient, t-distribution, and P values by Microsoft Excel.

## Results

### Trabectedin and olaparib synergism is related to PARP1 expression

**Fig. 1:**
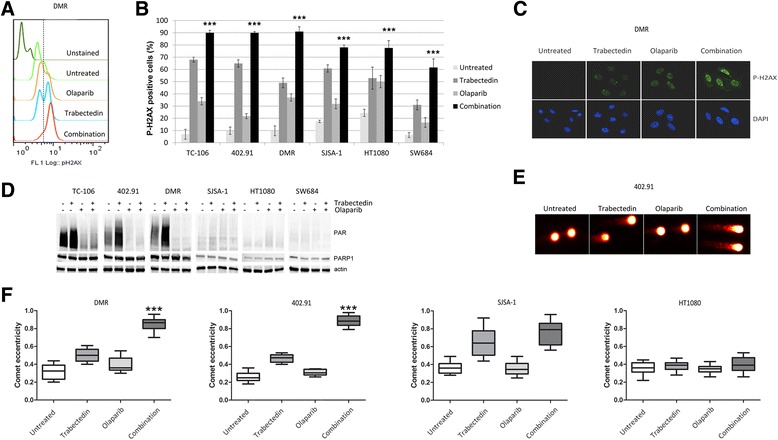
Olaparib enhanced trabectedin-induced DNA damage in high-PARP1-expressing cells. FACS analysis of P-H2AX positive cells after 24-h treatment with 0.125 nM trabectedin, 1.25 μM olaparib as single agents and in combination: **a** representative histograms of DMR cells and **b** mean quantification of P-H2AX in TC-106 (Ewing), 402.91 (liposarcoma), DMR (leiomyosarcoma), SJSA-1 (osteosarcoma), and HT1080 and SW684 (fibrosarcoma) cells; Y error bars indicate mean ± S.E.M;*** *p* < 0.001 between combination and both single agents and controls. **c** Immunofluorescence of P-H2AX in DMR cells treated as in (**a** and **b**); **d** Western blot analysis of PARP1 activity (PARylation) and PARP1 expression after 4-h treatment with 10 nM trabectedin, 20 μM olaparib as single agents and in combination; β-actin was done as loading control; **e-f** DNA fragmentation obtained after 48-h treatment with 0.125 nM trabectedin, 1.25 μM olaparib as single agents and in combination as revealed by COMET assay: E, representative photomicrographs of COMET tails in 402.91 cells; **f** Box-plot shows mean comet eccentricity ±25% (boxes) and the 5–95% percentile (whiskers)

Initially, we demonstrated that in bone and soft tissue sarcoma (BSTS) cell lines trabectedin treatment significantly increased phosphorylated histone H2AX (P-H2AX), the marker of DNA double-strand breaks (*p* < 0.001, Fig. 1a-c). Subsequently, we showed that after 24-h treatment with the combination of trabectedin (0.125 nM) and olaparib (1.25 μM) there was a variable but significant increase in P-H2AX positive cells if compared to single agents and untreated controls (Fig. 1a-c). Thereafter, we studied the role of PARP1 enzymatic activity (PARylation) after 4-h treatment with trabectedin (10 nM). We observed that in 3/6 (50%) bone and soft tissue sarcoma (BSTS) cell lines PARylation was highly increased, as compared to untreated controls (Fig. 1d). In these cells, the PARP1 inhibitor olaparib inhibited both basal and trabectedin-induced PARP1 activation remarkably well (Fig. 1d). To elucidate why trabectedin failed to activate PARP1 equally in all cell lines, we studied the correlation between PARP1 basal expression, activity and gene status. We found that after trabectedin exposure, PARylation was significantly increased in high- vs. low-PARP1-expressing cells (Fig. 1d). To further explain differences in PARylation activity, we performed mutational analysis of PARP1 gene and we found the Val762Ala single nucleotide polymorphism in SJSA-1 and SW684 cells (Additional file 2: Figure S2).

**Fig. 2:**
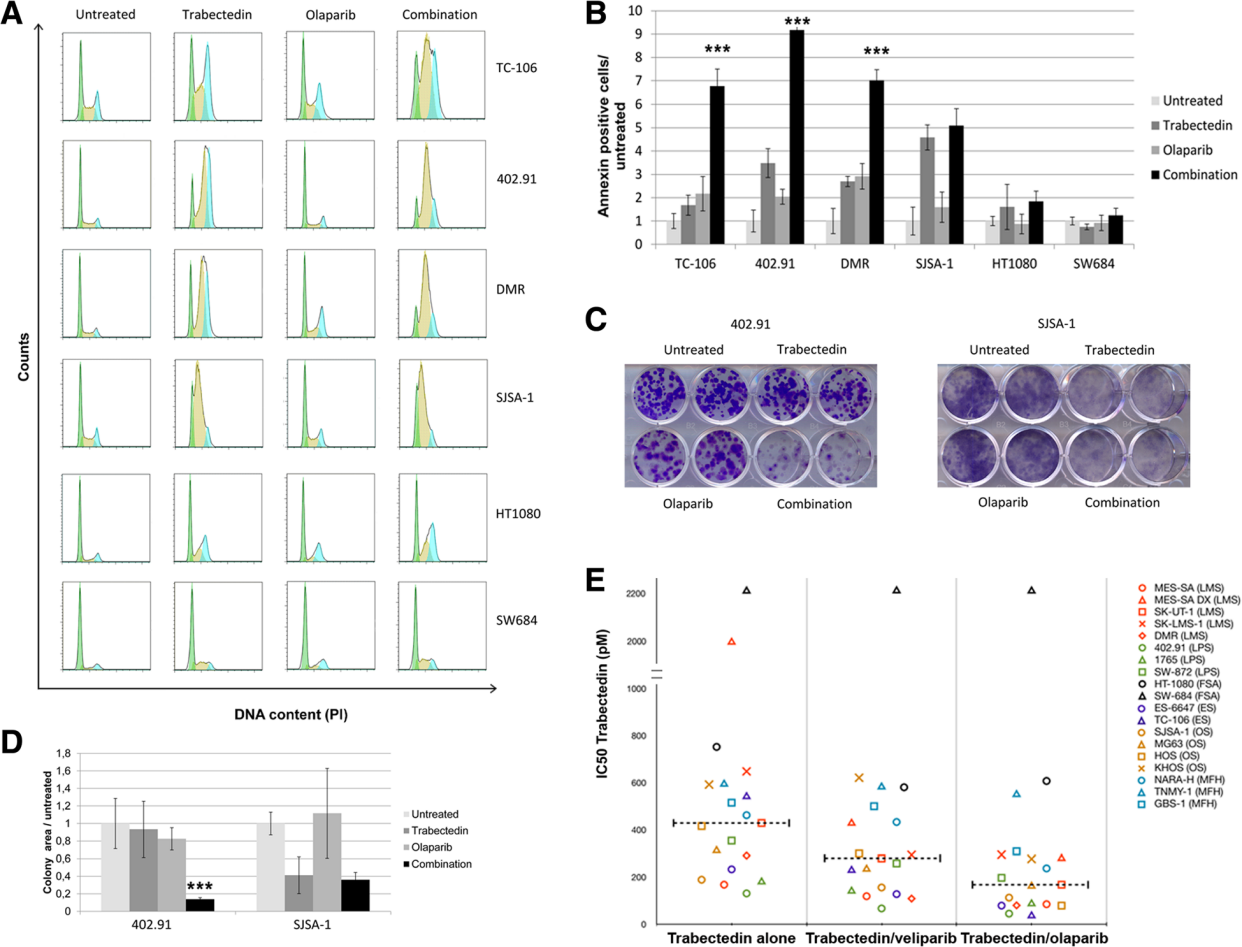
The synergistic antitumor activity of trabectedin and PARP inhibitors was cell line-dependent. FACS analysis of (**a**), DNA content (cell cycle distribution) after 48-h treatment with 0.125 nM trabectedin and 1.25 μM olaparib alone and their combination, *green, yellow*, and *light blue* represent G0/G1, S phase, and G2/M phase, respectively; as calculated by Flow Jo software; **b** apoptosis (Annexin V-PI staining) obtained after 72-h treatment with 0.125 nM trabectedin and 1.25 μM olaparib alone and their combination; **c** representative 7-day colony growth assay with 402.91 and SJSA-1 cells testing 0.125 nM trabectedin, 1.25 μM olaparib and their combination; **d** quantitation of three independent experiments of colony growth with 402.91 and SJSA-1 cells testing trabectedin, olaparib and their combination. **e** IC50 distribution of trabectedin as a single agent and in combination with olaparib or veliparib obtained by Calcusyn software after 72-h treatment with serial dilution of trabectedin (2–0.125 nM), olaparib (20–1.25 μM), veliparib (80–5 μM) as single agents or in constant combination with trabectedin, dashed lines indicate median IC50. Y error bars indicate mean ± S.E.M;*** *p* < 0.001 between combination and both single agents and controls

These results caused us to explore the effect of PARP1 inhibition on trabectedin-induced DNA damage and we found that 24-h treatment with the combination of trabectedin (0.125 nM) and olaparib (1.25 μM) significantly increased P-H2AX signaling in all cell lines tested (*p* < 0.001, Fig. 1a-c) if compared to both single agents and controls. Irreparable DNA fragmentation was significantly increased after 48-h treatment with the combination if compared to both single agents and controls in high-PARP1-expressing cells, as shown by COMET assay (*p* < 0.001; Fig. 1e-f). On the contrary, in low-PARP1-expressing cells, P-H2AX activation led to DNA repair. The intensity of the combination-induced DNA damage caused a cell line-dependent cell cycle arrest at G2/M checkpoint and the accumulation of cells into the late S phase (Fig. 2a), resulting in apoptosis after 96 h (Fig. 2b). This effect was consistently stronger in high-PARP1-expressing cells (TC-106, DMR, 402.91) and led to a significant inhibition of tumor cell colony growth (*p* < 0.001; Fig. 2c-d).

To expand our findings, we tested the antitumor activity of serial dilutions of two PARP1 inhibitors, olaparib (20–1.25 μM) and veliparib (80–5 μM) in a panel of 20 different BSTS cell lines, both as single agents and in constant combination with serial dilution of trabectedin (2–0.125 nM). We observed a strong synergism of trabectedin + olaparib in 18/20 cell lines while in the trabectedin + veliparib combination, we observed less synergism in 15/19 cell lines (Fig. 2e, Additional file 1: Table S1). In high-PARP1-expressing cells the trabectedin IC50s were significantly reduced if compared to low-PARP1-expressing cells both in combination with olaparib and veliparib (*p* = 0.01 and *p* = 0.02, respectively; Additional file 2: Figure S3).

Based on these in vitro experiments, we chose olaparib and trabectedin for further drug combination studies. As an example, Additional file 2: Figure S4 shows dose-effect curves obtained in TC-106, 402.91, DMR; SJSA-1, HT1080, and SW694 cells. Synergy was distributed along a spectrum of intensity; it was strongest in Ewing’s sarcoma cells and decreased progressively from liposarcoma (myxoid > dedifferentiated) to osteosarcoma to undifferentiated pleomorphic sarcoma to fibrosarcoma (Fig. 2e, Additional file 1: Table S1). Of note is that the doxorubicin-resistant leiomyosarcoma cell line MES-SA-DX5 also displayed cross-resistance to trabectedin, but addition of PARP1 inhibitors restored sensitivity to a clinically achievable trabectedin concentration (Additional file 1: Table S1 and Additional file 2: Figure S2e). Interestingly, MES-SA-DX5 cells displayed higher basal PARP1 expression and activity (Additional file 2: Figure S5 if compared with their parental counterparts, due to PARP1 gene amplification as shown by fluorescent in situ hybridization (FISH, Additional file 2: Figure S5 and Additional file 1: Table S4), real- time PCR (Additional file 1: Table S5), and by array comparative genomic hybridization (aCGH) analysis (https://www.ncbi.nlm.nih.gov/geo/query/acc.cgi?acc=GSE77175).

### Cell cycle control and DNA-repair pathways are involved in the activity of trabectedin and olaparib combination

**Fig. 3:**
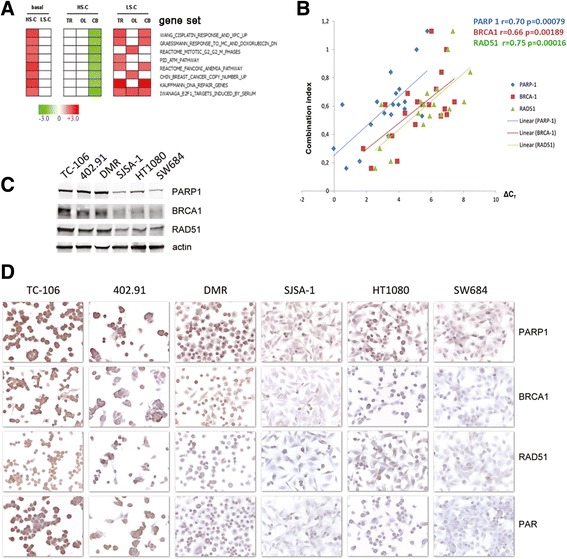
PARP1, RAD51, and BRCA1 expression and gene signatures as predictive markers of trabectedin and olaparib synergism. **a**, enrichment analysis of gene sets (GSEA) published in Molecular Signatures Database v5.0 of Broad Institute obtained by comparing mRNA expression profiles in cells displaying high combination synergism (HS-C: TC-106; DMR; 402.91) with cells displaying low/no synergy (LS-C: SJSA-1; HT1080; SW684) at basal condition (untreated) or after 24-h treatment with 1.25 nM trabectedin (TR), 1.25 μM olaparib (OL), or combination (CB); **b**, direct correlation (linear regression) between synergism of trabectedin and olaparib in combination (combination index) and mRNA expression (ΔC_T_) of PARP1, BRCA1, and RAD51 in 19 human sarcoma cell lines (as in Additional file 1: Table S3); Pearson score r and *P* value are indicated in *blue*, *red* and *green* for PARP1, BRCA1 and RAD51 respectively; **c**, western blot analysis and D, immunocytochemistry of PARP1, BRCA1, and RAD51 expression in TC-106, DMR, 402.91, SJSA-1, HT1080, and SW684

To dissect the molecular mechanism behind trabectedin + olaparib synergism, we compared the gene expression profiles (https://www.ncbi.nlm.nih.gov/geo/query/acc.cgi?acc=GSE76981) of three cell lines with high synergism (HS-C) and three cell lines with low/no synergism (LS-C). First, we compared gene expression profiles of HS-C and LS-C by gene set enrichment analysis (GSEA) to identify gene sets associated with the sensitive phenotype and we found 437 gene sets matching the criteria of false discovery rate (FDR) <0.05 and we challenged them with GSEA, comparing each treated cells against untreated. Of these ones, 57 gene sets were specifically enriched in combination treatment in HS-C, but not in single treatments. Of note, the expression of specific gene sets involved in DNA-damage response, DNA-repair pathways, and cell cycle control were higher in HS-C than in LS-C (Fig. 3a). Notably, these gene sets were specifically down-modulated by the drug combination-not by single agents- in HS-C, but were either unmodified or upregulated in LS-C (Fig. 3a). Taken together, these results indicated that specific gene sets involved in the DNA-damage response, G2/M cell cycle checkpoint, and DNA-repair pathways were implicated in the mechanisms behind trabectedin/olaparib synergism.

Consistent evidences were shown also by comparing genomic profiles of HS-C and LS-C as obtained by aCGH (Additional file 2: Figure S6, Additional file 3 and https://www.ncbi.nlm.nih.gov/geo/query/acc.cgi?acc=GSE77175). Differential aberration analysis demonstrated four groups of genomic alterations that clearly distinguished HS-C from LS-C (Additional file 3). Namely, regions of chromosomes enriched in genes involved in DNA-damage response/repair and G2/M cell cycle checkpoint were deleted and amplified in HS- and LS-cells, respectively (as highlighted in Additional file 3).

To validate these findings, we confirmed the genomic status of selected genes (Additional file 1: Table S4-S6) with different approaches (FISH, real-time PCR on genomic DNA, multiplex ligation-dependent probes amplification, denaturing high performance liquid chromatography, and sequencing) and analyzed expression of DNA-damage response/repair pathways key components in 19 cell lines using quantitative real-time PCR (Additional file 1: Table S3). Our results show that mRNA expression of PARP1, RAD51, and BRCA1 (but not their genomic status) is directly related to combination synergism (Fig. 3b, Additional file 1: Table S2-S5). Furthermore, in selected experiments, we confirmed these data at protein level by western blot and immunocytochemistry, and demonstrated that the expression of PARP1, BRCA1, and RAD51 was higher in HS-C than in LS-C (Fig. 3c-d).

**Fig. 4:**
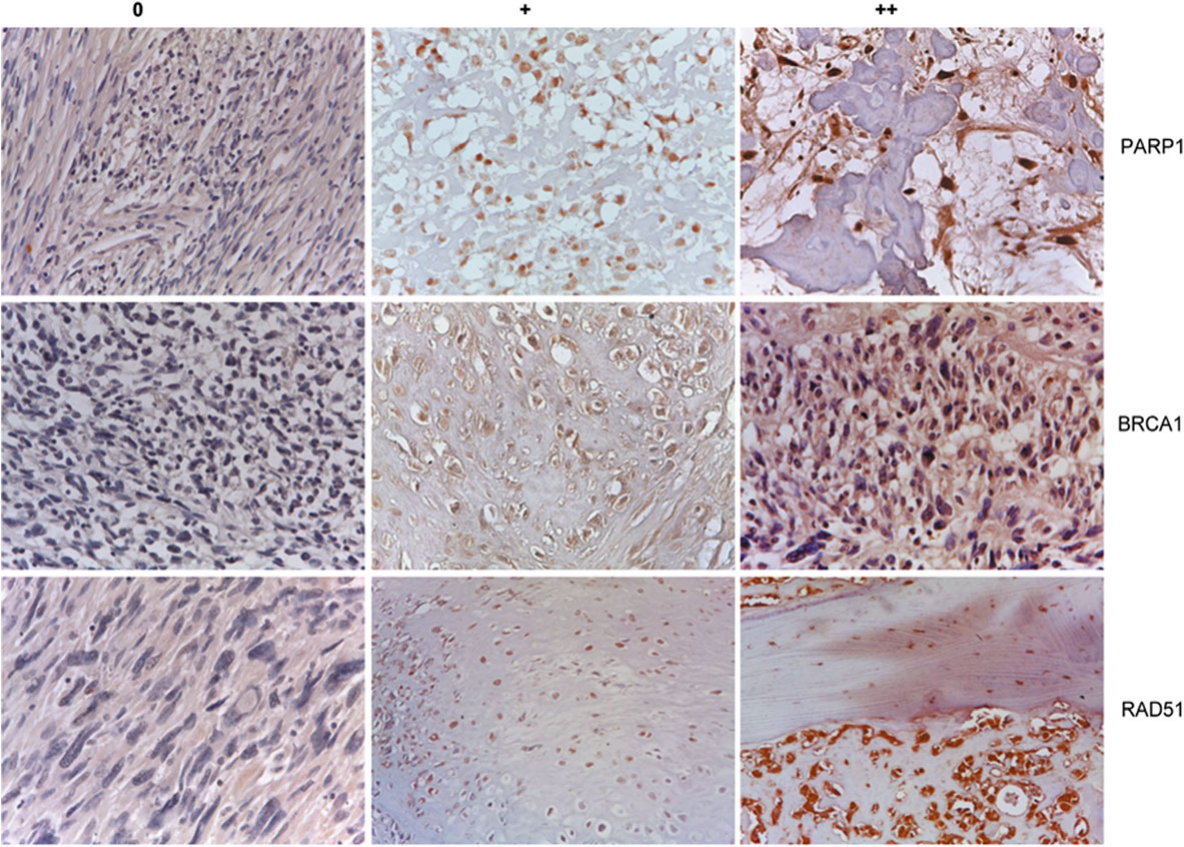
Representative immunohistochemical analysis and relative Intensity Scores (0, +, ++) of PARP1, BRCA1, and RAD51 expression in patient-derived sarcoma specimens

Next, we evaluated the expression of these putative biomarkers in patients who might benefit from trabectedin + olaparib treatment. We employed immunohistochemistry (IHC) to measure PARP1, BRCA1, and RAD51 expression in a panel of 54 patient-derived sarcoma specimens. PARP1, BRCA1, and RAD51 were overexpressed in 34/53 (64%), 18/54 (33%), and 35/54 (65%) samples, respectively (Fig. 4, Additional file 1: Table S7). For all, IHC expression was characterized by high concordance rates (Additional file 1: Table S8) with an 85% (95% CI: 73–97%) and 86% (95% CI: 75–97%) probability of finding PARP1 overexpressed in BRCA1- and RAD51-positive samples, respectively.

### Antitumor activity of trabectedin and olaparib combination in HS-C and LS-C mice models

Thereafter, we studied in vivo antitumor activity of the combination. We subcutaneously injected (s.c.) one tumorigenic HS-C (DMR) and one LS-C (SJSA-1) in nonobese diabetic/sever combined immunodeficient (NOD/SCID) mice. Mice with tumors ≥32 mm^3^ were randomized to receive drug treatment.

**Fig. 5:**
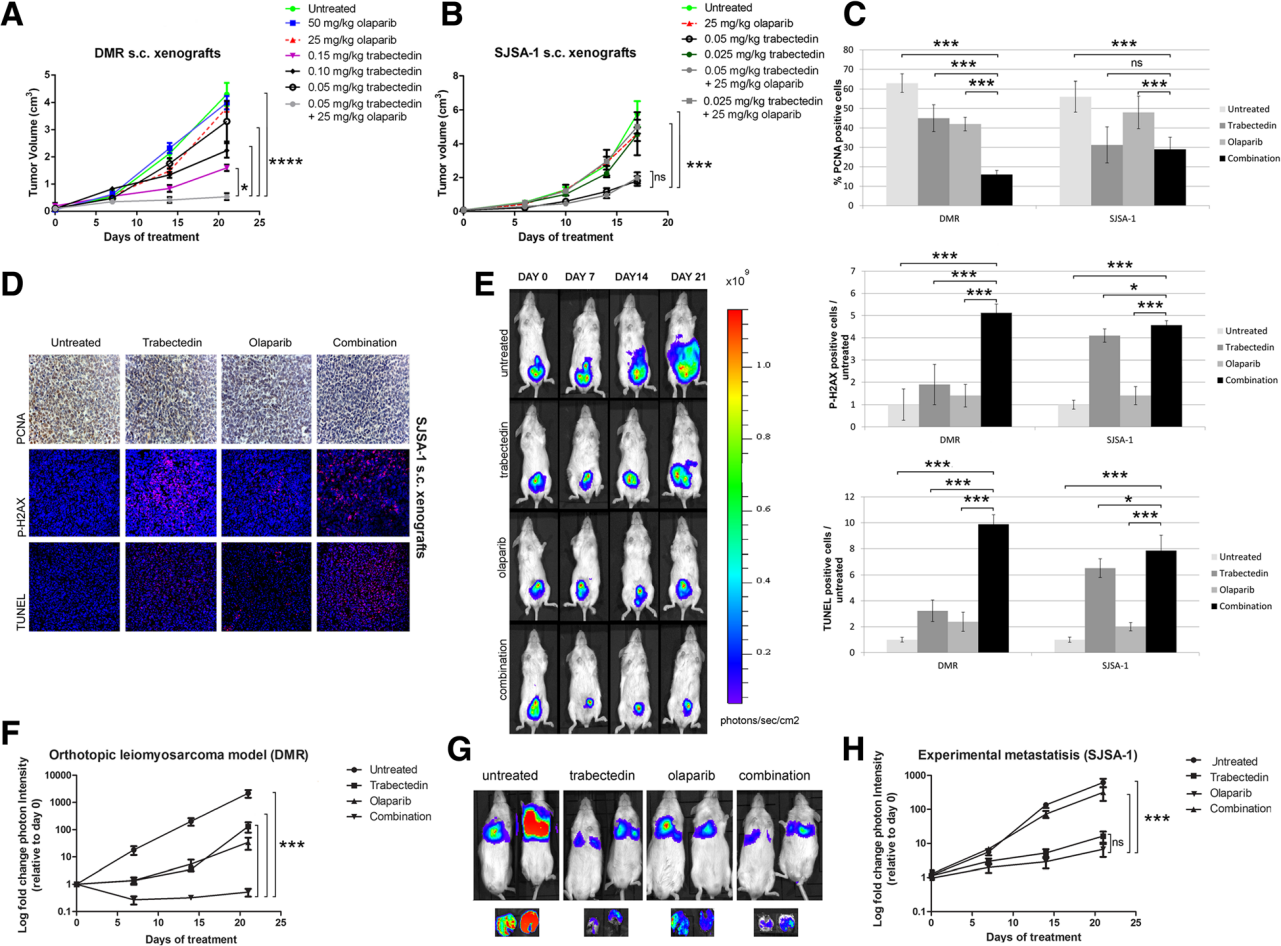
Trabectedin and olaparib combination showed antitumor effects in in vivo models. **a-b**, Tumor volume and **c-d**, histological analysis of proliferating cells (PCNA staining), DNA damage (P-H2AX), and apoptosis (TUNEL) in DMR and SJSA-1 s.c. xenografted NOD/SCID mice treated with trabectedin and olaparib, as single agent and in combination or untreated. **e-f**, in vivo imaging of tumor growth and spread in orthotopic uterine leiomyosarcoma model and **g-h**, tumor colony growth in e.v. SJSA xenografted NOD/SCID mice treated with trabectedin and olaparib as single agents and in combination, or left untreated; in vivo imaging was done 3 days after the end of the 21-day- treatment; Y error bars indicate mean ± S.E.M; *p < 0.05; **p < 0.01; *** *p* < 0.001

In DMR s.c. model, mean tumor volume was significantly reduced by the treatment with two doses of trabectedin (0.15 and 0.1 mg/kg, *p* < 0.0001) and not by the lowest dose (0.05 mg/kg; ns, non-significant, p > 0.05). Olaparib as single agent (two doses, 50 and 25 mg/kg/day) did not show antitumor activity. Interestingly, the combination of trabectedin and olaparib at the lowest concentrations (25 mg/kg/day olaparib + 0.05 mg/kg trabectedin) significantly reduced tumor growth if compared to untreated controls and each single agent (Fig. 5a). In SJSA-1 s.c. models the treatment with 0.05 mg/kg of trabectedin significantly reduced tumor growth and no further advantage was obtained by the combination with 25 mg/kg/day of olaparib (ns, p > 0.05) (Fig. 5b). At the lowest dosage (0.025 mg/kg) trabectedin did not impinge tumor growth both as single agent and in combination with olaparib 25 mg/kg (Fig. 5b). Tumor cell proliferation was evaluated by PCNA staining (Fig. 5c), DNA damage by P-H2Ax foci formation and apoptosis by TUNEL staining in combination-treated mice and compared to both controls and single agents (0.05 mg/kg trabectedin and 25 mg/kg olaparib). In DMR s.c. models, cell proliferation was significantly reduced (PCNA, Fig. 5c), and consistently both DNA damage (P-H2AX, Fig. 5c) and apoptosis (TUNEL, Fig. 5c) were significantly increased by the combination if compared to single agents and controls. Evaluation of the same parameters in LS-C SJSA-1 xenografts showed that trabectedin was effective as single agent, and only a marginal advantage was obtained by the combination (Fig. 5c-d). The combination of trabectedin and olaparib did not cause evident signs of toxicity after gross necropsy or weight loss during treatment.

To assess combination activity in the metastatic setting, we developed an orthotopic model of uterine leiomyosarcoma with HS-C DMR cells and an experimental metastatic model of osteosarcoma with LS-C SJSA-1 cells. DMR cells injected into uterine wall tissue developed local and distant metastases within 3 weeks in the untreated control group. Treatment with single agents delayed, but did not impede, tumor progression, whereas trabectedin + olaparib not only significantly reduced primary tumor growth, but also prevented metastatic spread (*p* < 0.001 vs. single agents and control; Fig. 5e-f). On the contrary, in the metastatic osteosarcoma model, the combination showed non-significant advantage over trabectedin alone (p > 0.05) (Fig. 5g-h). These in vivo experiments confirm data observed in vitro, wherein low-PARP1-expressing cells consistently demonstrated lower synergy of the combination.

### PARP1 expression dictates the response to trabectedin and olaparib combination

**Fig. 6:**
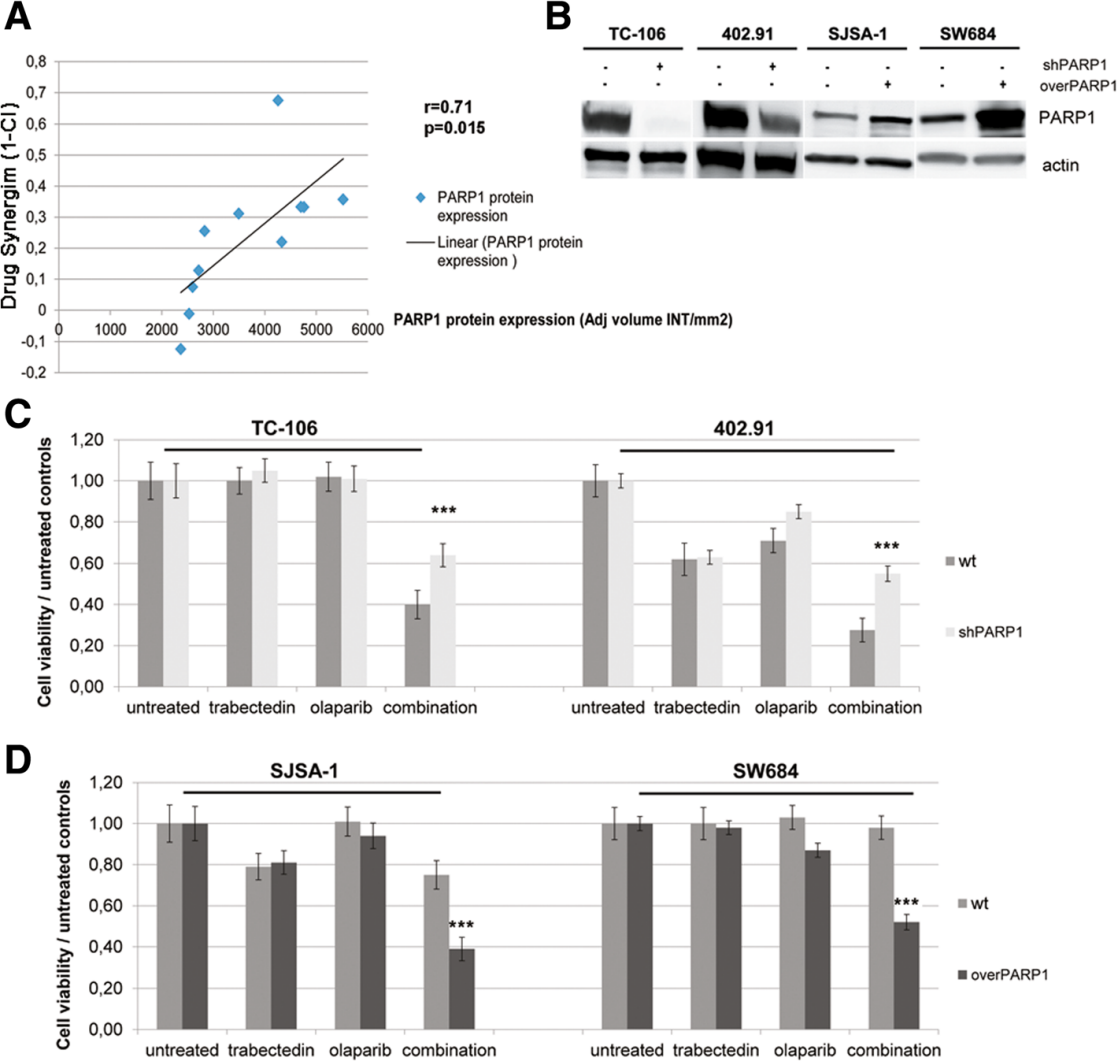
**a** direct correlation (linear regression) between drug synergism of trabectedin and olaparib in combination (1 - combination index) and PARP1 protein expression (western blot image quantitation) in 11 human tumor cell lines with different origin (Additional file 1: Table S2); Pearson score r and *P* value are indicated; **b** western blot analysis of PARP1 and actin (as housekeeping) protein expression in cells stably transduced with lentiviral vector carrying PARP1 specific shRNA for silencing or functional PARP1 gene for overexpression; **c** cell viability assay in PARP1 stably silenced (shPARP1) HS-C TC-106 and 402.91 cells and their parental wild type (wt) counterparts; **d** cell viability assay in LS-C (SJSA-1 and SW684) overexpressing PARP1 (overPARP1) and their parental wild type (wt), Y error bars indicate mean ± S.E.M; *** *p* < 0.001 between combination and both single agents and controls

To generalize to other tumor settings our observations on PARP1 inhibition, we tested trabectedin + olaparib activity in an independent panel of 11 tumor cell lines of different origins (prostate, lung, bile duct, breast), and verified a direct correlation between PARP1 protein expression and combination synergy (Fig. 6a, Additional file 1: Table S2). Finally, the functional role of PARP1 to determine trabectedin + olaparib synergy was further proved in silencing and overexpression experiments. Namely, PARP1 expression was downregulated with specific transient and stable silencing (Fig. 6b) in two HS-C (TC-106, 402.91), which resulted in significantly reduced trabectedin + olaparib activity compared to mock-treated cells (*p* < 0.001; Fig. 6c). On the other hand, the overexpression of PARP1, determined by stable lentiviral transduction (Fig. 6b) in two LS-C (SJSA-1, SW684), consistently and significantly increased combination activity vs. mock-treated controls (*p* < 0.001; Fig. 6d). Indeed, activity rose to levels observed in HS-C (Fig. 6c-d).

### PARP1 expression determines the cellular response to different DNA-damaging agents

Finally, to generalize the key role played by PARP1 basal expression in synergism, we asked whether enzyme basal expression might drive tumor cell response to other cytotoxics used in combination with PARP1 inhibitors. We investigated PARylation in both low- vs. high-PARP1-expressing cells and PARP1-silenced vs. wild-type counterparts (Additional file 2: Figure S7A) after the treatment with different chemotherapeutic agents: cisplatin, dacarbazine, actinomycin-D, etoposide, doxorubicin, and gemcitabine characterized by different mechanism of action: crosslinking, methylation, transcription inhibition, topoisomerase II inhibition, DNA intercalation, and nucleotide mimicking, respectively. Consistently with trabectedin experiments, we found that PARylation correlated directly with PARP1 basal expression (higher in high- vs. low-PARP1-expressing cells), regardless of the studied cytotoxic or its mechanism of action (Additional file 2: Figure S7B). Indeed, in MSTO-H211 parental cells cisplatin, etoposide, gemcitabine and trabectedin were potent activator of PARP1 activity, while doxorubicin, actinomycin-D and dacarbazine (as pro-drug) less efficiently induced PAR (Additional file 2: Figure S7B). After PARP1 silencing in MSTO-H211 cells, PARylation was invariably decreased if compared to parental cells after each treatment.

## Discussion

We identified and demonstrated that PARP1 basal expression is crucial in the mechanism behind the synergy between PARP1 inhibitors and trabectedin independent of BRCA1/2 status, in several robust models across different histotypes. The chemo-sensitization observed in high-PARP1-expressing cells suggests that PARP1 inhibitors may extend and improve the clinical application of DNA-repair targeting when combined with other cytotoxics also.

Our findings support a reappraisal of PARP1 inhibitors as chemo-sensitizers. In general, after chemotherapy exposure, tumor cell fate is highly dependent on the efficiency of the same DNA-damage response pathways that restore normal cell DNA integrity. This observation fueled the idea to target key points in DNA-repair machinery to increase cytotoxicity [2, 4] and brought about the development of several compounds, among which PARP1 inhibitors eventually demonstrated clinical efficacy [4]. However, so far, their clinical use is limited to tumors harboring hereditary or acquired genetic defects in homologous recombination (HR) exploiting an intrinsic cell weakness through so-called “synthetic lethality” [28–34]. Interestingly, patients affected by HR-defective tumors have the greatest benefit from treatment with DNA-damaging agents, such as platinum compounds and trabectedin [37, 38, 47].

We pursued to induce a sort of “chemical synthetic lethality” also in HR-proficient tumors, by combining trabectedin with PARP1inhibitors. Our hypothesis stemmed from the observation that trabectedin not only induced a peculiar DNA damage but also caused impairment in the DNA-repair machinery that might become lethal in presence of PARP1 inhibitors [35].

Demonstration that trabectedin strongly activates PARP1 enzyme strengthened our idea to combine this chemotherapy with PARP1 inhibitors. However, after trabectedin treatment, we observed significantly different PARylation across various histotypes. For example, single nucleotide polymorphism in the PARP1 gene (Val762Ala) is known to result in a less efficient PARP1 variant [48, 49], which may partly explain differences in the degree of PARylation. To understand the impact of PARylation differences, we turned to PARP1 basal expression and proved that PARP1 protein basal level was not only directly proportional to chemotherapy-induced PARP1 activation, but also that it dictated the synergism with trabectedin and several other chemotherapy compounds as well. Indeed, PARP1-depleted cells were tolerant to PARP1 inhibitors [50, 51], and therefore, PARP1 activity is the prerequisite to induce a significant amount of complexes formed by PARP1, damaged DNA and PARP1 inhibitor that are plausibly more cytotoxic than unrepaired single-strand breaks alone [52–54]. Clearly, trabectedin synergized with both tested PARP1 inhibitors, but we found olaparib was significantly more potent than veliparib. Our result reflects the fact that veliparib is a pure catalytic inhibitor of PARP1 activation, whereas olaparib is a poisoning drug that blocks activated PARP1 enzyme at the site of damage (PARP1-trapping activity) impeding the subsequent recruitment of repairing machinery [11, 52, 55]. We studied the synergism of PARP1 inhibitors and trabectedin in a large set of different tumor cell lines and in in vivo models, demonstrating that PARP1 inhibition improved the antitumor activity of trabectedin in a cell-line dependent intensity, as previously shown in breast cancer and in Ewing’s sarcoma cell lines [44, 55]. Among the tested BSTS histotypes, Ewing’s sarcoma cells were the most sensitive to the combination, a result that might be explained by the known exquisite sensitivity of the pathognomonic fusion protein EWS/FLI1-expressing cells to both trabectedin and PARP1 inhibition [10, 56–59].

Another important datum emerged from study of the MES-SA-DX5 model. This leiomyosarcoma cell line had been previously made resistant to doxorubicin [60], and interestingly, we found it also shared cross-resistance to trabectedin. Of note is that the addition of PARP1 inhibitors restored sensitivity to clinically achievable concentration of trabectedin. The increased activity in MES-SA-DX5 can be explained by comparing the genomic and protein status of this cell line with its parental counterpart. Indeed in MES-SA-DX5, the PARP1 gene was amplified and consequently caused higher PARP1 expression and activity that confirmed the mechanistic role played by PARP1 in determining synergy between trabectedin and PARP1 inhibitors. These consistent observations further prove the key role played by PARP1 expression.

Given the redundant complexity of DNA repair machinery, we took advantage of gene array analyses to delve deeper into the mechanisms behind trabectedin + olaparib synergism. We identified specific gene sets involved in the combination synergism. These genes belong to cell cycle control such as normally activated G2/M cell cycle checkpoints and DNA-damage response and repair pathways (i.e., Timeless, AURKA, MCM2, RAD54L, POLD1, MRE11A) [61–64]. Moreover, specific genomic aberrations of these genes differentially characterized HS-C and LS-C. We showed that enzymes involved in DNA-damage response and repair were actually expressed more in cell lines displaying higher PARP1 basal expression and high trabectedin + olaparib synergism. The cell cycle phase is crucial for trabectedin and olaparib response and these findings confirmed our data on cell cycle perturbation. Undeniably, trabectedin-induced DNA damage activates the DNA-damage response leading to G2/M cell cycle arrest [35]. However, in this phase, repairing enzymes may still fix the damage caused by either single agent, such that tumor cells survive after initial cell cycle arrest. On the contrary, when the combination was used, DNA repair and cell survival were impeded because of irreparable DNA fragmentation and apoptosis.

Again the intensity of this event directly correlated with PARP1 expression. We carefully validated the role of identified candidate genes in two independent panels of 20 and 11 different cell lines. We selected key enzymes of DNA-damage response and repair pathways and investigated their expression by real-time PCR in the first 20 BSTS cell-line panel. We confirmed a statistically significant direct correlation between PARP1 expression and trabectedin + olaparib synergism. Interestingly, we also observed a significant correlation between drug synergism and RAD51 and BRCA1 expression, which might be explained by the fact that in HR-proficient cells, PARP1 expression drives the transcription of BRCA1 and RAD51 genes co-activating E2F transcription factor [65–69]. If so, then the statistical correlation between combination synergism and RAD51 or BRCA1 expression might simply reflect the level of PARP1 expression. These data are consistent with previous observations of correlation between DNA repair protein expression and sensitivity to PARP1 inhibition [70]. Nonetheless, in HR-defective cells PARP1 activity was found increased [54]. However, we did not find any correlation between the genomic status of key DNA repair genes and the synergism of the combination. To strengthen our observation and generalize our hypothesis on the role of PARP1 expression also in non-sarcoma cells, we replicated our experiments in a second independent panel (11 cell lines of different tumor origin) and confirmed the correlation between trabectedin + olaparib synergy and PARP1 expression also at the protein level. The functional role of PARP1 was further validated in silencing and overexpression experiments that confirmed the data reported above. Noteworthy, the modulation of PARP1 expression or the inhibition of its activity might modulate the nuclear factor Kappa-b pathway that is a crucial determinant of drug resistance [71–73]. This intriguing aspect warrants further investigations.

## Conclusions

Our data demonstrated that trabectedin is an ideal partner of PARP1 inhibitors because it potently activates PARP1. Furthermore, PARP1 basal expression drives the synergism intensity between PARP1 inhibitors and trabectedin. Indeed, the crucial role of PARP1 expression was confirmed in several BSTS histotypes and in other cancer types as well. Further studies of combinations of PARP1 inhibitors and other cytotoxics should consider basal PARP1 expression and activation after each drug exposure. Finally, olaparib and trabectedin combination is particularly attractive in tumors harboring high PARP1 expression and a specific DNA-damage response/repair and cell cycle control gene signatures that might become predictive biomarkers of response. Our findings pave the way for the future clinical study of trabectedin + olaparib combination independent of BRCA1/2 status.

## Additional files

Additional file 1: Table S1. Concentrations inhibiting 50% of cell viability (IC50) after 72- h treatment with serial dilutions of trabectedin (2–0.125 nM), olaparib (20–1.25 μM), and veliparib (80–5 μM) as single agents (a) or in constant combination (b, c), and their 95% confidence intervals (95% CI). Population doubling time. Combination index ± estimated standard deviation (est st dev) calculated at IC50 by the Chou-Talalay method for trabectedin and olaparib in combination and trabectedin and veliparib in combination in cell lines of different histotypes: leiomyosarcoma (LMS), undifferentiated pleomorphic sarcoma (UPS), myxoid liposarcoma (LMS), dedifferentiated liposarcoma (DLS), fibrosarcoma (FSA), synovial sarcoma (SS), Ewing’s sarcoma (ES), and osteosarcoma (OS). Supplier and culture conditions are reported for each cell line. **Table S2**. Direct correlation between combination index (calculated at IC50) and PARP1 protein intensity expressed as Pearson score (r Pearson). IC50 and 95% confidence intervals (95% CI) were calculated after 72-h treatment with serial dilutions of trabectedin (2–0.125 nM), olaparib (20–1.25μM), and their constant combination. Cell line characteristics, population doubling time, purchasers and culture conditions were included. **Table S3**. Gene expression (ΔCT) of DNA-damage response and repair key components and drug synergism expressed by combination index (CI). The correlation between each gene expression and the CI was evaluated by Pearson score (r); t distribution and their relative P value were shown. Yellow cells highlight significant direct correlation. **Table S4**. PARP1 gene (chromosome 1 q42.12d) copy number obtained by FISH. **Table S5**. PARP1, BRCA1, RAD51 gene copy number obtained by real- time PCR on genomic DNA. The gene copy number of PARP1, RAD51, and BRCA1 did not correlate with the Combination index (CI) as shown by Pearson score. **Table S6**. Genomic status of selected genes analyzed by MLPA and DHPLC /Sequencing. Red cells indicate increased copy number, while blue cells indicate reduced copy number as obtained by DHPLC analysis. **Table S7**. Immunohistochemistry score of intensity for PARP1, BRCA1, and RAD51 protein expression in formalin-fixed paraffin-embedded sarcoma samples. **Table S8**. 2 × 2 contingency tables of immunohistochemistry (IHC) expression of PARP1, BRCA1, and RAD51 in patient-derived soft tissue and bone sarcoma specimens (a, b, c) and related concordance rates (d). (DOCX 2086 kb)

Additional file 2: Figure S1. Overview of gene expression analysis. GSEA, gene signature enrichment analysis. **Figure S2**. DNA sequences of single nucleotide polymorphism at codon 762 of PARP1 gene in HT1080, SJSA-1, and SW684 cells. **Figure S3**. Distribution of trabectedin IC50 as single agent (TR alone) and in combination with veliparib (TR + VEL) or olaparib (TR + OL) among high-PARP1-expressing cells (red triangle) and low-PARP1-expressing cells (blue triangle). **Figure S4**. Dose- response curve obtained after 72-h treatment with trabectedin (2–0.125nM), olaparib (20–1.25 μM) as single agents and in constant combination. **Figure S5**. A, western blot analysis of PARylation and PARP1 expression in MES-SA and MES-SA-DX5 leiomyosarcoma cells; B, FISH analysis of PARP1 gene (red) and centromere of chromosome 1 (green) in MESSA and MESSA-DX5. **Figure S6**. Genomic status as obtained by aCGH analysis of TC-106, 402.91, DMR, SJSA-1, HT1080, SW684: gain (red) and loss (green) of chromosome regions. **Figure S7**. A, Western blot analysis of PARylation and PARP1 expression and B, quantitation of PAR in MSTO-H211, and PARP1-silenced MSTO-H211 untreated or treated with 10nM trabectedin, 20 μM cisplatin (Sandoz), 20 μM gemcitabine (Sandoz), 20 μM doxorubicin (Pfizer), 20 μM dacarbazine (Medac), 20 μM etoposide (Teva), 50 mM actinomycin-D (Thermo Fisher Scientific), β-actin was done as loading control. (DOCX 5982 kb)

Additional file 3: Analysis of differential genomic aberrations in HS-C and LS-C cells. (PDF 255 kb)

## Abbreviations

aCGH: Array comparative genomic hybridization
Ala: Alanine
ATM: Ataxia telangiectasia mutated
AURKA: Aurora kinase A
BRCA1/2: Breast Cancer Type 1/2 susceptibility protein
BSTS: Bone and soft tissue sarcoma
CHEK2: Checkpoint kinase 2
DHPLC: Denaturing high performance liquid chromatography
DNA: Deoxyribonucleic acid
EWS: Ewing sarcoma protein
FDR: False discovery rate
FISH: Fluorescent in situ hybridization
FLI1: Friend leukemia integration 1 transcription factor
GO: Gene ontology
GSEA: Gene set enrichment analysis
HS-C: High synergism- cells
i.v.: Intravenous
LS-C: Low synergism cells
MCM2: Minichromosome maintenance protein 2
MLPA: Multiplex ligation-dependent probes amplification
MRE11A: Homolog of meiotic recombination 11, S. cerevisiae
ns: Non-significant
PARP1: Poly-ADP-ribosyl-transferase-1
PARylation: Poly- ADP- ribosylation
PCNA: Proliferating cell nuclear antigen
PCR: Polymerase chain reaction
P-H2AX: Phosphorylated histone H2AX
PI: Propidium iodide
POLD1: Polymerase (DNA-directed) delta 1
PTEN: Phosphatase and tensin homolog
RAD54L: RAD54-Like protein
s.c.: Subcutaneous
STR: Short tandem repeat
TC-NER: transcription-coupled nucleotide excision repair
TUNEL: Terminal deoxynucleotidyl transferase dUTP nick end labeling
Val: Valine
