# Supplementary material for: PARP1 expression drives the synergistic antitumor activity of trabectedin and PARP1 inhibitors in sarcoma preclinical models
Source: Mol Cancer. 2017 Apr 28;16:86. doi: 10.1186/s12943-017-0652-5 (PMC5410089; doi:10.1186/s12943-017-0652-5)
Supplement: Supplementary file 1 — Concentrations inhibiting 50% of cell viability (IC50) after 72- h treatment with serial dilutions of trabectedin (2–0.125 nM), olaparib (20–1.25 μM), and veliparib (80–5 μM) as single agents (a) or in constant combination (b, c), and their 95% confidence intervals (95% CI). Population doubling time. Combination index ± estimated standard deviation (est st dev) calculated at IC50 by the Chou-Talalay method for trabectedin and olaparib in combination and trabectedin and veliparib in combination in cell lines of different histotypes: leiomyosarcoma (LMS), undifferentiated pleomorphic sarcoma (UPS), myxoid liposarcoma (LMS), dedifferentiated liposarcoma (DLS), fibrosarcoma (FSA), synovial sarcoma (SS), Ewing’s sarcoma (ES), and osteosarcoma (OS). Supplier and culture conditions are reported for each cell line. Table S2. Direct correlation between combination index (calculated at IC50) and PARP1 protein intensity expressed as Pearson score (r Pearson). IC50 and 95% confidence intervals (95% CI) were calculated after 72-h treatment with serial dilutions of trabectedin (2–0.125 nM), olaparib (20–1.25μM), and their constant combination. Cell line characteristics, population doubling time, purchasers and culture conditions were included. Table S3. Gene expression (ΔCT) of DNA-damage response and repair key components and drug synergism expressed by combination index (CI). The correlation between each gene expression and the CI was evaluated by Pearson score (r); t distribution and their relative P value were shown. Yellow cells highlight significant direct correlation. Table S4. PARP1 gene (chromosome 1 q42.12d) copy number obtained by FISH. Table S5. PARP1, BRCA1, RAD51 gene copy number obtained by real- time PCR on genomic DNA. The gene copy number of PARP1, RAD51, and BRCA1 did not correlate with the Combination index (CI) as shown by Pearson score. Table S6. Genomic status of selected genes analyzed by MLPA and DHPLC /Sequencing. Red cells indicate increased copy number [file 12943_2017_652_MOESM1_ESM.docx]

**Additional file Tables**

**Paper title:** PARP1 expression drives the synergistic antitumor activity of trabectedin and PARP1 inhibitors in sarcoma preclinical models.

**Authors:** Ymera Pignochino, Federica Capozzi, Lorenzo D'Ambrosio, Carmine Dell'Aglio, Marco Basiricò, Marta Canta, Annalisa Lorenzato, Francesca Vignolo Lutati, Sandra Aliberti, Erica Palesandro, Paola Boccone, Danilo Galizia, Sara Miano, Giulia Chiabotto, Lucia Napione, Loretta Gammaitoni, Dario Sangiolo, Maria Serena Benassi, Giovanna Chiorino, Barbara Pasini, Massimo Aglietta, Giovanni Grignani.

**Table S1.** Concentrations inhibiting 50% of cell viability (IC50) after 72- hour treatment with serial dilutions of trabectedin (2-0.125 nM), olaparib (20-1.25 µM), and veliparib (80-5 µM) as single agents (a) or in constant combination (b, c), and their 95% confidence intervals (95%CI). Population doubling time. Combination index ± estimated standard deviation (est st dev) calculated at IC50 by the Chou-Talalay method for trabectedin and olaparib in combination and trabectedin and veliparib in combination in cell lines of different histotypes: leiomyosarcoma (LMS), undifferentiated pleomorphic sarcoma (UPS), myxoid liposarcoma (LMS), dedifferentiated liposarcoma (DLS), fibrosarcoma (FSA), synovial sarcoma (SS), Ewing’s sarcoma (ES), and osteosarcoma (OS). Supplier and culture conditions are reported for each cell line.

**
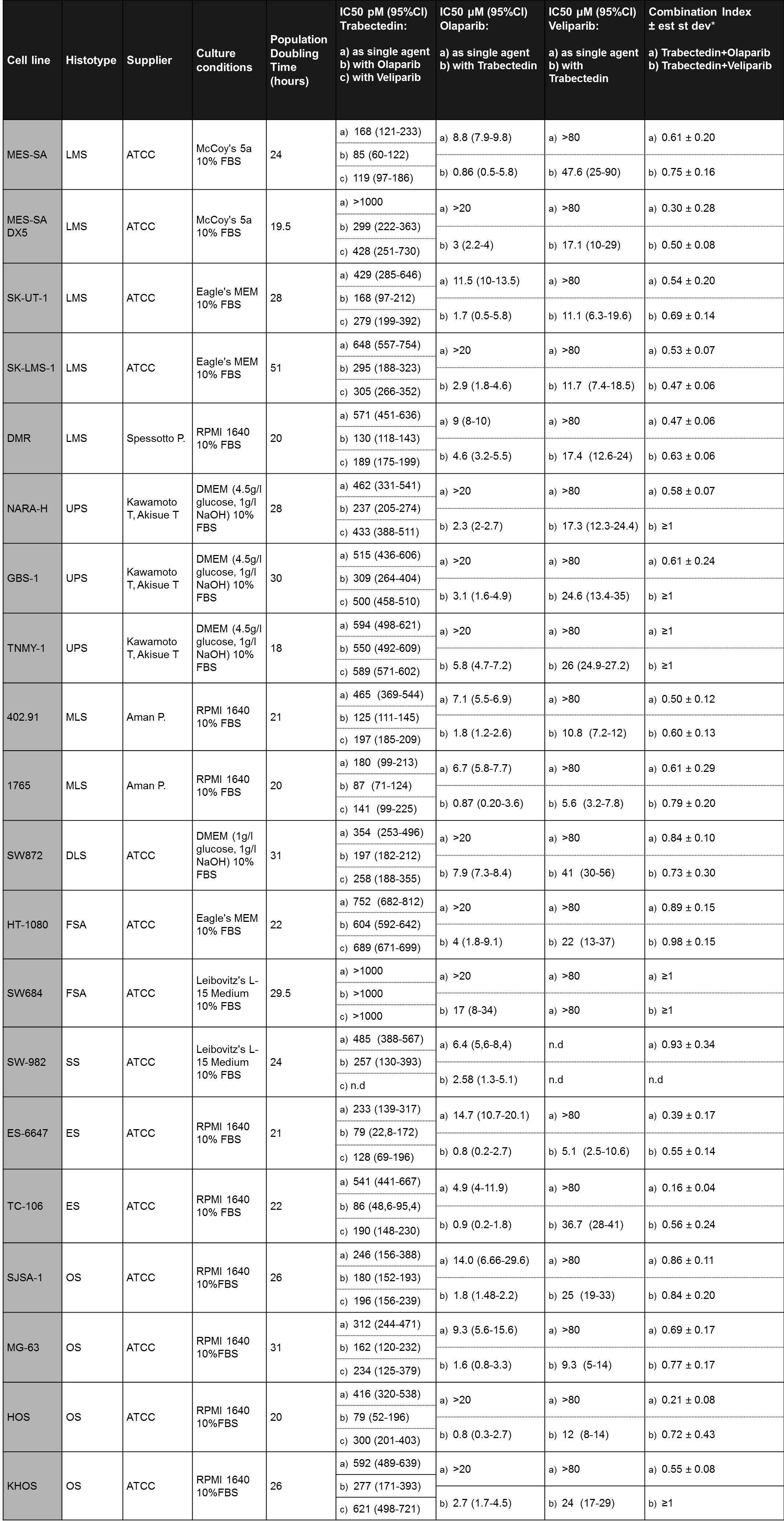
**

**Table S2.** Direct correlation between combination index (calculated at IC50) and PARP1 protein intensity expressed as Pearson score (r Pearson). IC50 and 95% confidence intervals (95%CI) were calculated after 72-h treatment with serial dilutions of trabectedin (2-0.125 nM), olaparib (20-1.25µM), and their constant combination. Cell line characteristics, population doubling time , purchasers and culture conditions were included.

**Table S3.** Gene expression (ΔC_T_) of DNA-damage response and repair key components and drug synergism expressed by combination index (CI). The correlation between each gene expression and the CI was evaluated by Pearson score (r); t distribution and their relative P value were shown. Yellow cells highlight significant direct correlation.


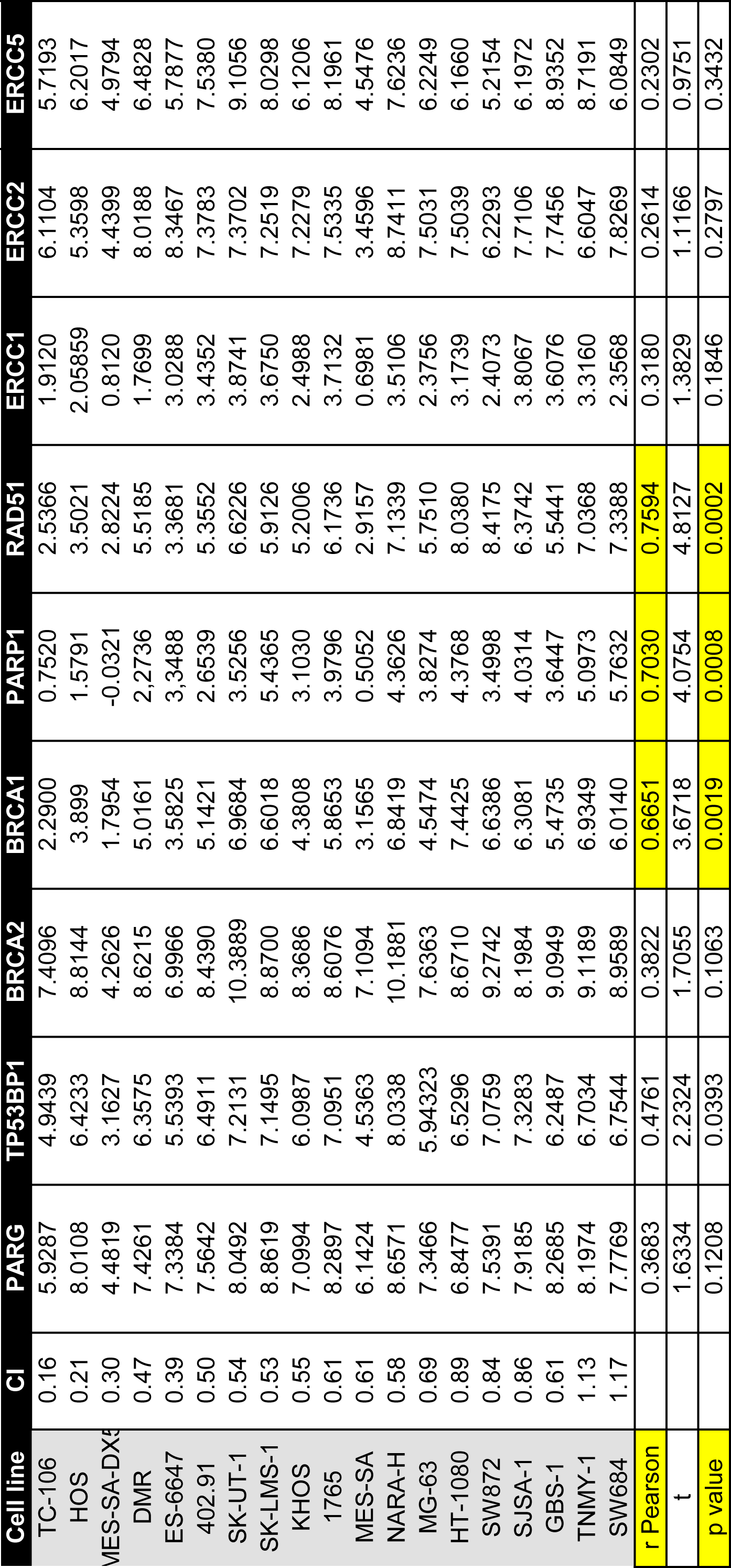


**Table S4.** PARP1 gene (chromosome 1 q42.12d) copy number obtained by FISH.

| **Cell line** | **PARP1 ( copy number /cell)** |
| --- | --- |
| TC-106 | 2 |
| 402.91 | 2 |
| DMR | 3 ( triploid) |
| Sjsa-1 | 2 |
| HT1080 | 2 |
| Sw684 | 4 |
| Nara-H | 2 |
| MES-SA | 2 |
| MES-SA/DX5 | 4 |

**Table S5.** PARP1, BRCA1, RAD51 gene copy number obtained by real- time PCR on genomic DNA. The gene copy number of PARP1, RAD51, and BRCA1 did not correlate with the Combination index (CI) as shown by Pearson score.


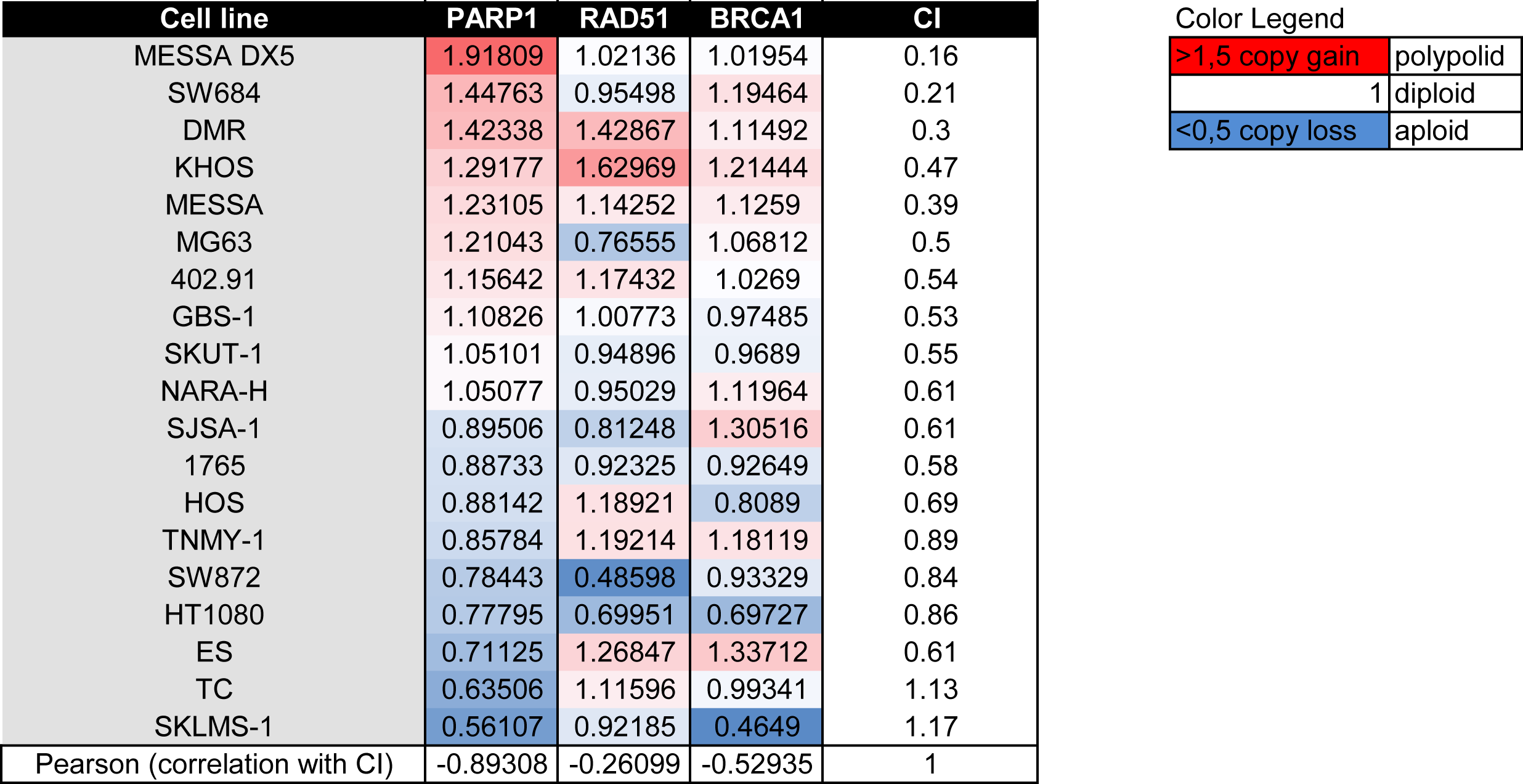


**Table S6.** Genomic status of selected genes analyzed by MLPA and DHPLC /Sequencing. Red cells indicate increased copy number, while blue cells indicate reduced copy number as obtained by DHPLC analysis.


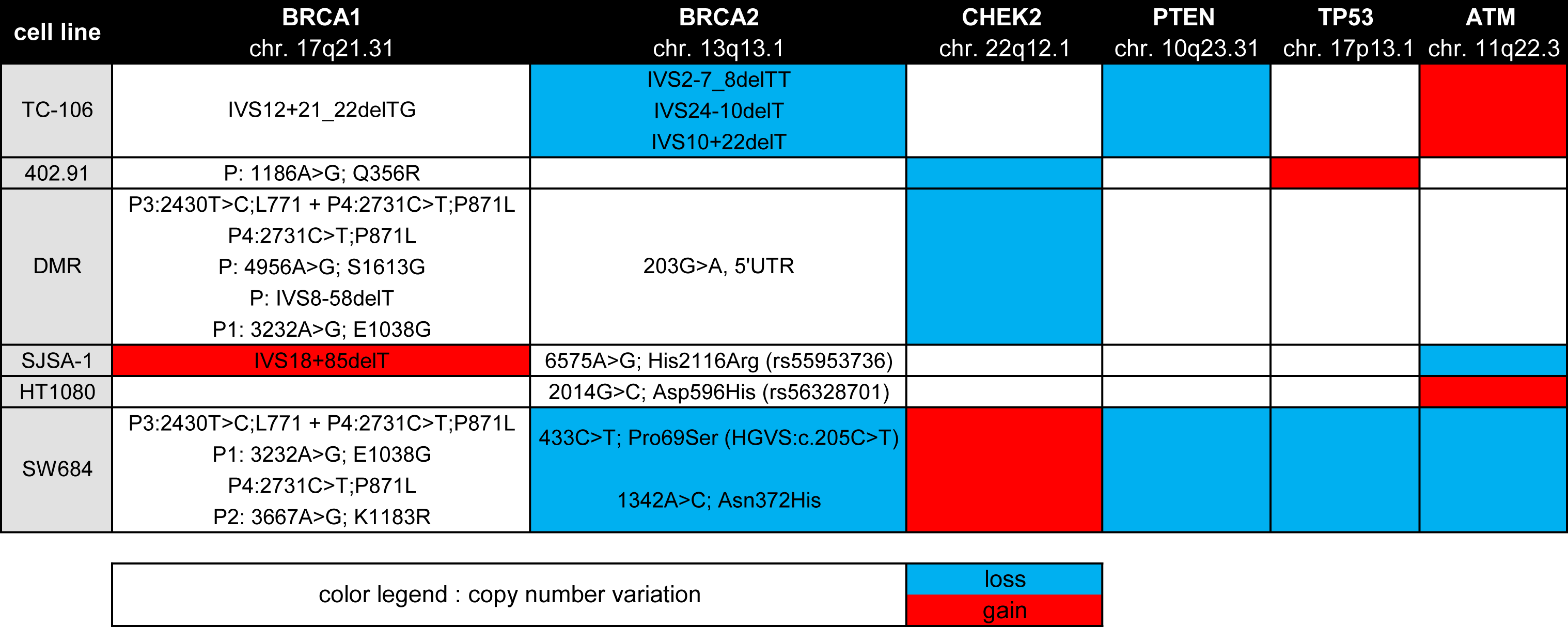


**Table S7.** Immunohistochemistry score of intensity for PARP1, BRCA1, and RAD51 protein expression in formalin-fixed paraffin-embedded sarcoma samples.

**
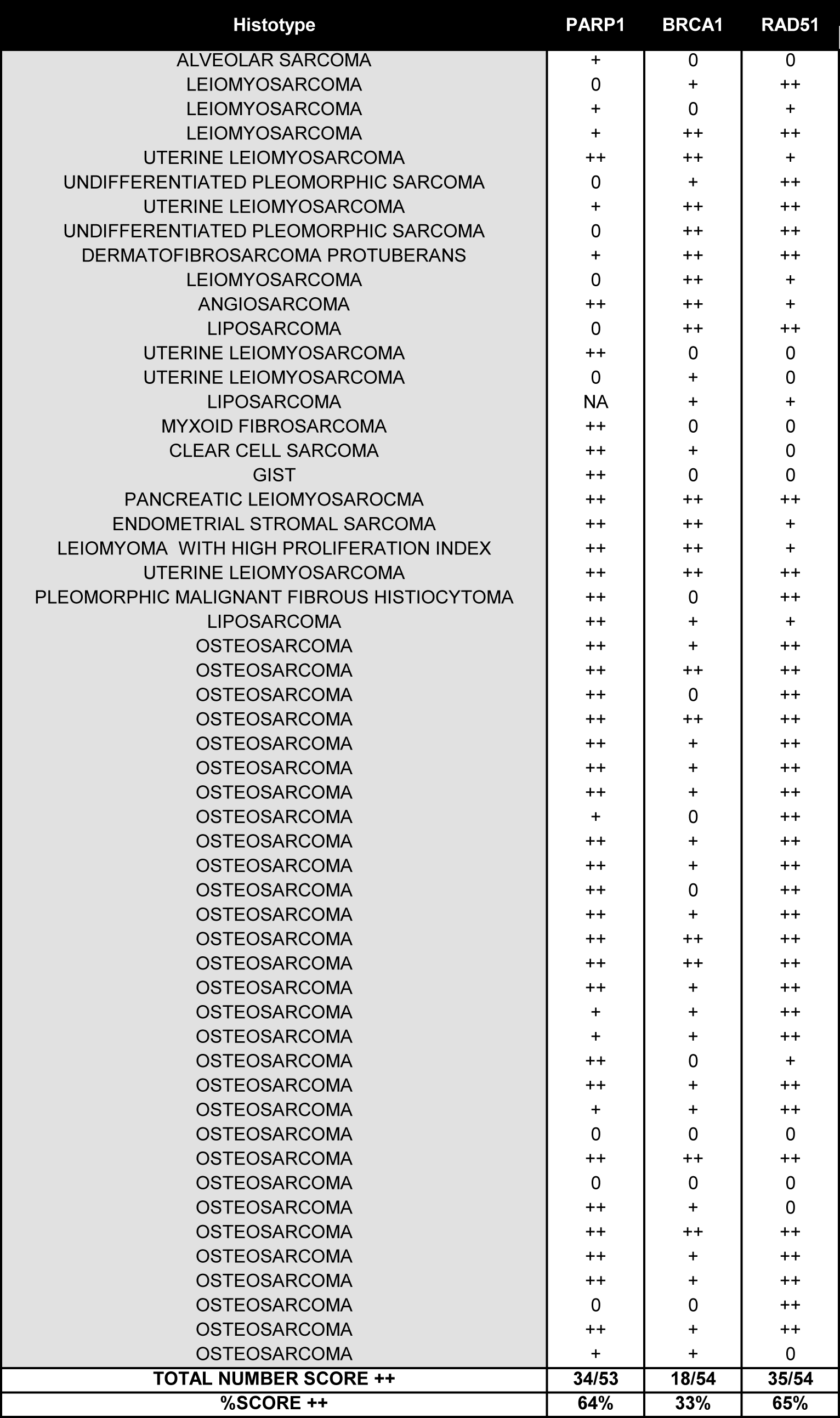
**

**Table S8.** 2x2 contingency tables of immunohistochemistry (IHC) expression of PARP1, BRCA1, and RAD51 in patient-derived soft tissue and bone sarcoma specimens (**a, b, c**) and related concordance rates (**d**).

| **A** | | | **BRCA1 expression** | | **Total** |
| --- | --- | --- | --- | --- | --- |
|  |  |  | **-** | **+** |  |
| **PARP1 expression** | **-** | **Count** | 3 | 6 | 9 |
|  |  | **% within PARP1 expression** | 33.3% | 66.7% | 100.0% |
|  |  | **% within BRCA1 expression** | 23.1% | 15.0% | 17.0% |
|  |  | **% of total** | 5.7% | 11.3% | 17.0% |
|  | **+** | **Count** | 10 | 34 | 44 |
|  |  | **% within PARP1 expression** | 22.7% | 77.3% | 100.0% |
|  |  | **% within BRCA1 expression** | 76.9% | 85.0% | 83.0% |
|  |  | **% of total** | 18.9% | 64.2% | 83.0% |
| **Total** | | **Count** | 13 | 40 | 53 |
|  |  | **% within PARP1 expression** | 24.5% | 75.5% | 100.0% |
|  |  | **% within BRCA1 expression** | 100.0% | 100.0% | 100.0% |
|  |  | **% of total** | 24.5% | 75.5% | 100.0% |

| **B** | | | **RAD51 expression** | | **Total** |
| --- | --- | --- | --- | --- | --- |
|  |  |  | **-** | **+** |  |
| **PARP1 expression** | **-** | **Count** | 3 | 6 | 9 |
|  |  | **% within PARP1 expression** | 33.3% | 66.7% | 100.0% |
|  |  | **% within RAD51 expression** | 30.0% | 14.0% | 17.0% |
|  |  | **% of total** | 5.7% | 11.3% | 17.0% |
|  | **+** | **Count** | 7 | 37 | 44 |
|  |  | **% within PARP1 expression** | 15.9% | 84.1% | 100.0% |
|  |  | **% within RAD51 expression** | 70.0% | 86.0% | 83.0% |
|  |  | **% of total** | 13.2% | 69.8% | 83.0% |
| **Total** | | **Count** | 10 | 43 | 53 |
|  |  | **% within PARP1 expression** | 18.9% | 81.1% | 100.0% |
|  |  | **% within RAD51 expression** | 100.0% | 100.0% | 100.0% |
|  |  | **% of total** | 18.9% | 81.1% | 100.0% |

| **C** | | | **RAD51 expression** | | **Total** |
| --- | --- | --- | --- | --- | --- |
|  |  |  | **-** | **+** |  |
| **BRCA1 expression** | **-** | **Count** | 6 | 7 | 13 |
|  |  | **% within BRCA1 expression** | 46.2% | 53.8% | 100.0% |
|  |  | **% within RAD51 expression** | 60.0% | 15.9% | 24.1% |
|  |  | **% of total** | 11.1% | 13.0% | 24.1% |
|  | **+** | **Count** | 4 | 37 | 41 |
|  |  | **% within BRCA1 expression** | 9.8% | 90.2% | 100.0% |
|  |  | **% within RAD51 expression** | 40.0% | 84.1% | 75.9% |
|  |  | **% of total** | 7.4% | 68.5% | 75.9% |
| **Total** | | **Count** | 10 | 44 | 54 |
|  |  | **% within BRCA1 expression** | 18.5% | 81.5% | 100.0% |
|  |  | **% within RAD51 expression** | 100.0% | 100.0% | 100.0% |
|  |  | **% of total** | 18.5% | 81.5% | 100.0% |

**D. Concordance rates of IHC expression of biomarkers**

| **PARP1-BRCA1** | 69.8% |
| --- | --- |
| **PARP1-RAD51** | 75.5% |
| **BRCA1-RAD51** | 79.6% |
