# Supplementary material for: PARP1 expression drives the synergistic antitumor activity of trabectedin and PARP1 inhibitors in sarcoma preclinical models
Source: Mol Cancer. 2017 Apr 28;16:86. doi: 10.1186/s12943-017-0652-5 (PMC5410089; doi:10.1186/s12943-017-0652-5)
Supplement: Supplementary file 2 — Overview of gene expression analysis. GSEA, gene signature enrichment analysis. Figure S2. DNA sequences of single nucleotide polymorphism at codon 762 of PARP1 gene in HT1080, SJSA-1, and SW684 cells. Figure S3. Distribution of trabectedin IC50 as single agent (TR alone) and in combination with veliparib (TR + VEL) or olaparib (TR + OL) among high-PARP1-expressing cells (red triangle) and low-PARP1-expressing cells (blue triangle). Figure S4. Dose- response curve obtained after 72-h treatment with trabectedin (2–0.125nM), olaparib (20–1.25 μM) as single agents and in constant combination. Figure S5. A, western blot analysis of PARylation and PARP1 expression in MES-SA and MES-SA-DX5 leiomyosarcoma cells; B, FISH analysis of PARP1 gene (red) and centromere of chromosome 1 (green) in MESSA and MESSA-DX5. Figure S6. Genomic status as obtained by aCGH analysis of TC-106, 402.91, DMR, SJSA-1, HT1080, SW684: gain (red) and loss (green) of chromosome regions. Figure S7. A, Western blot analysis of PARylation and PARP1 expression and B, quantitation of PAR in MSTO-H211, and PARP1-silenced MSTO-H211 untreated or treated with 10nM trabectedin, 20 μM cisplatin (Sandoz), 20 μM gemcitabine (Sandoz), 20 μM doxorubicin (Pfizer), 20 μM dacarbazine (Medac), 20 μM etoposide (Teva), 50 mM actinomycin-D (Thermo Fisher Scientific), β-actin was done as loading control. (DOCX 5982 kb) [file 12943_2017_652_MOESM2_ESM.docx]

**Additional file Figures**

**Paper title:** PARP1 expression drives the synergistic antitumor activity of trabectedin and PARP1 inhibitors in sarcoma preclinical models

**Authors:** Ymera Pignochino, Federica Capozzi, Lorenzo D'Ambrosio, Carmine Dell'Aglio, Marco Basiricò, Marta Canta, Annalisa Lorenzato, Francesca Vignolo Lutati, Sandra Aliberti, Erica Palesandro, Paola Boccone, Danilo Galizia, Sara Miano, Giulia Chiabotto, Lucia Napione, Loretta Gammaitoni, Dario Sangiolo, Maria Serena Benassi, Giovanna Chiorino, Barbara Pasini, Massimo Aglietta, Giovanni Grignani.

**Figure S1.** Overview of gene expression analysis. GSEA, gene signature enrichment analysis.


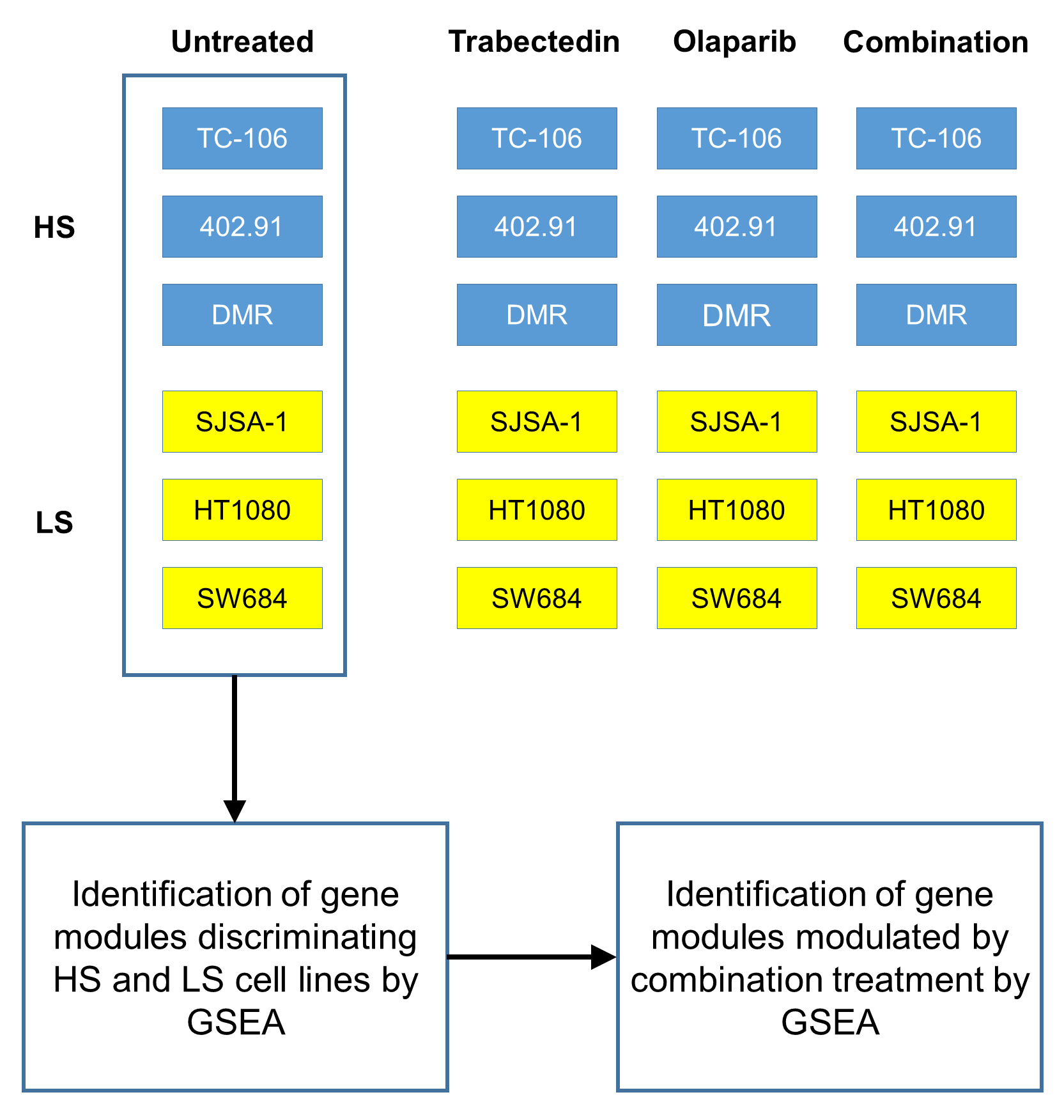


**Figure S2.** DNA sequences of single nucleotide polymorphism at codon 762 of PARP1 gene in HT1080, SJSA-1, and SW684 cells.

**Figure S3.** Distribution of trabectedin IC50 as single agent (TR alone) and in combination with veliparib (TR+VEL) or olaparib (TR+OL) among high-PARP1-expressing cells (red triangle) and low-PARP1-expressing cells (blue triangle).


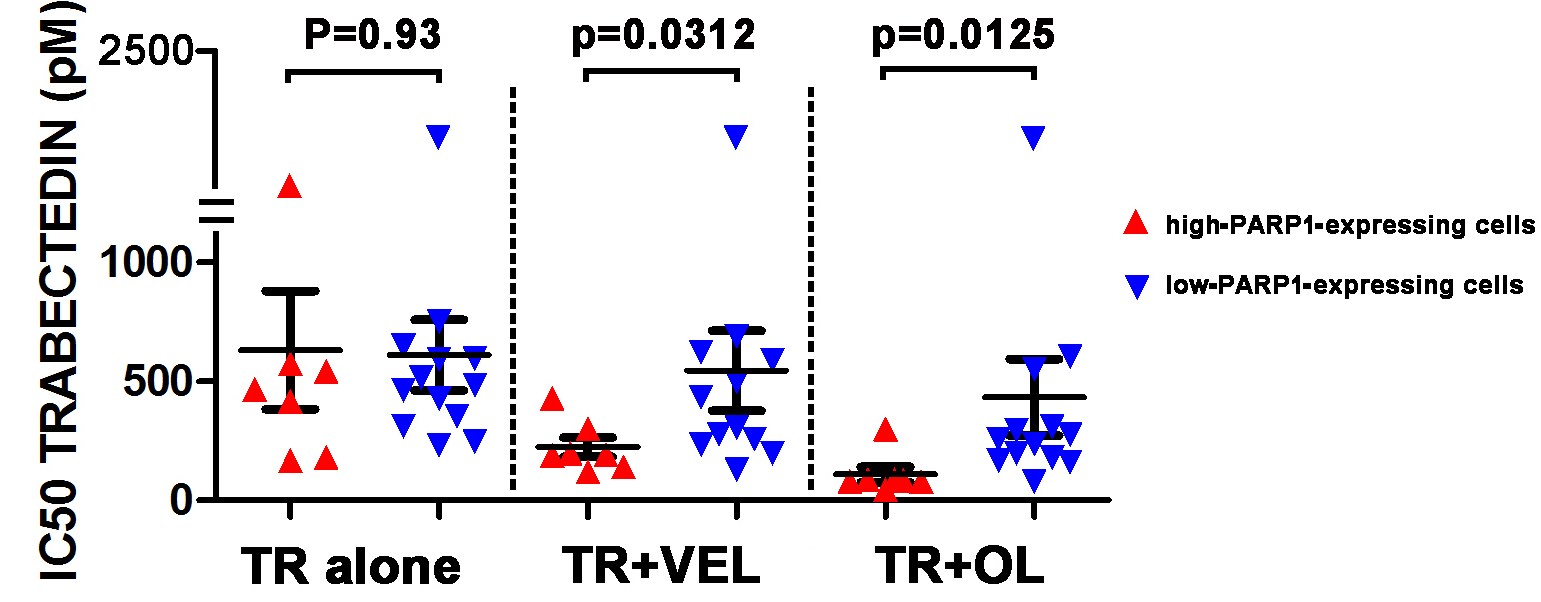


MES-SA DX5

MES-SA DX5

MES-SA DX5

SW684

SW684

SW684

**Figure S4.** Dose- response curve obtained after 72-h treatment with trabectedin (2-0.125nM), olaparib (20-1.25 µM) as single agents and in constant combination.


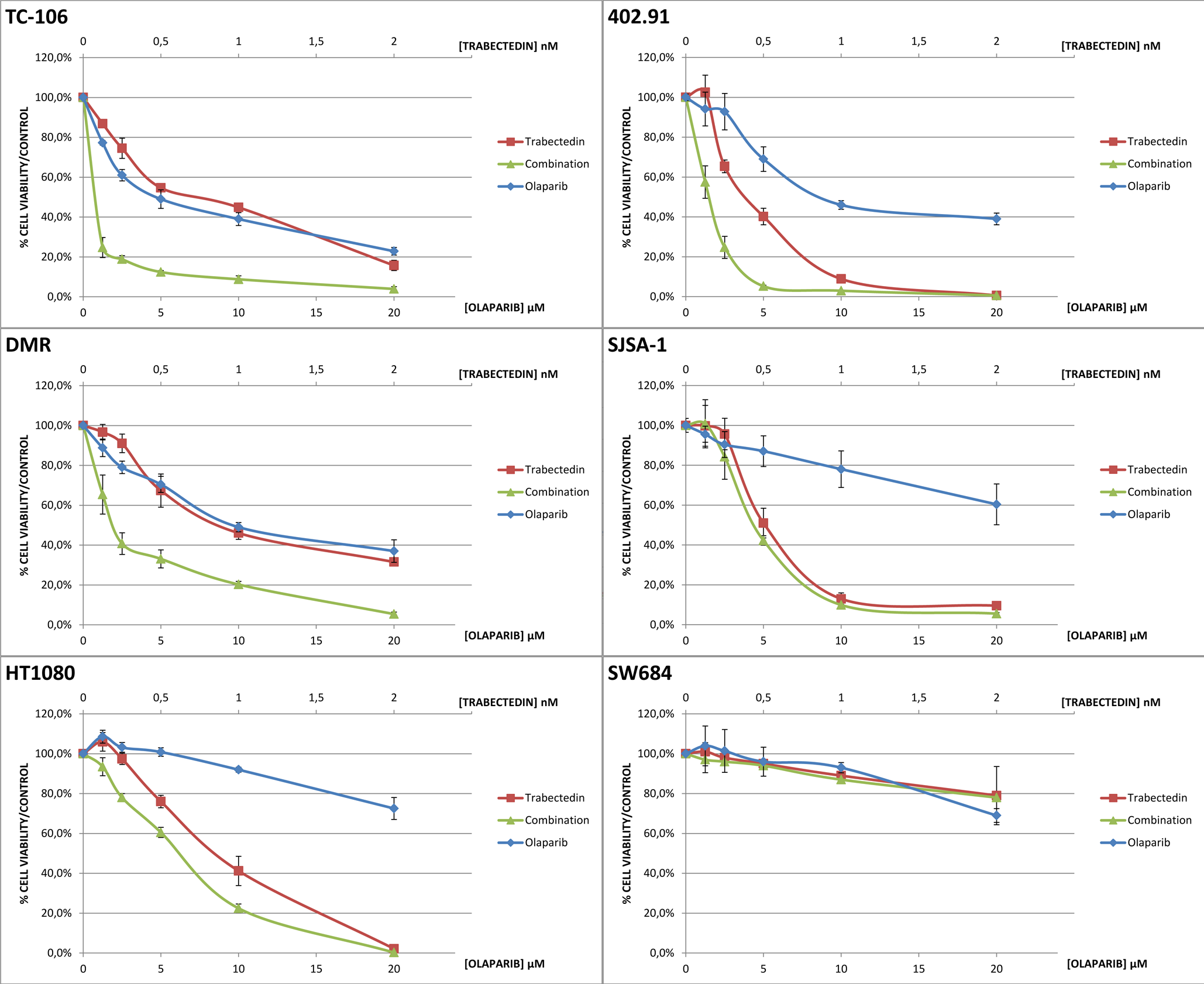


**Fig. S5.** A, western blot analysis of PARylation and PARP1 expression in MES-SA and MES-SA-DX5 leiomyosarcoma cells; B, FISH analysis of PARP1 gene (red) and centromere of chromosome 1 (green) in MESSA and MESSA-DX5.

**
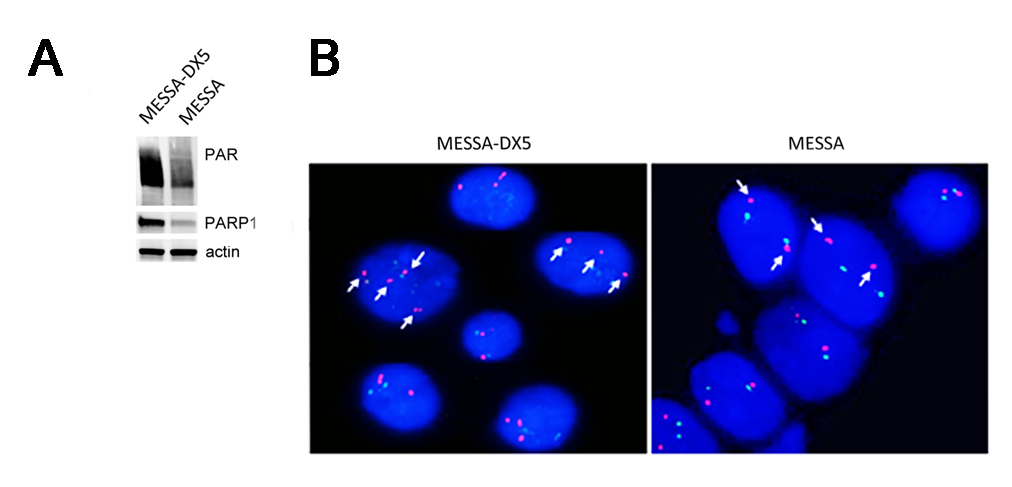
**

**Fig. S6.** Genomic status as obtained by aCGH analysis of TC-106, 402.91, DMR, SJSA-1, HT1080, SW684: gain (red) and loss (green) of chromosome regions

**Suppl. Fig S7.** A, Western blot analysis of PARylation and PARP1 expression and B, quantitation of PAR in MSTO-H211, and PARP1-silenced MSTO-H211 untreated or treated with 10nM trabectedin, 20 µM cisplatin (Sandoz), 20 µM gemcitabine (Sandoz), 20 µM doxorubicin (Pfizer), 20 µM dacarbazine (Medac), 20 µM etoposide (Teva), 50 mM actinomycin-D (Thermo Fisher Scientific), β-actin was done as loading control.

**
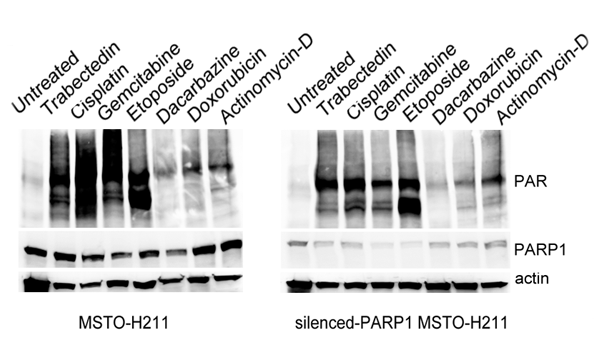
**

**B**

**A**

**
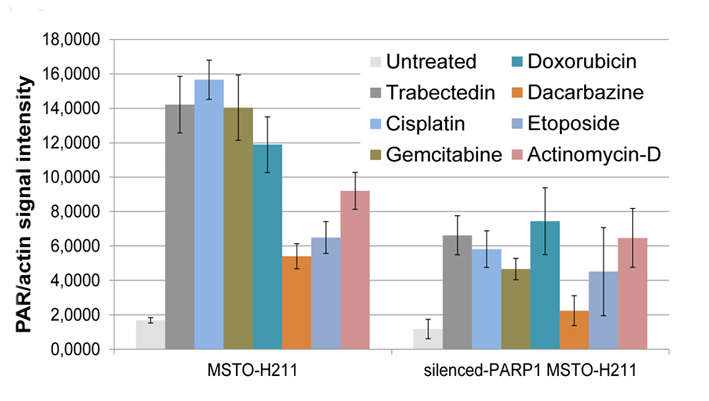
**
