# Supplementary material for: PARP1 expression drives the synergistic antitumor activity of trabectedin and PARP1 inhibitors in sarcoma preclinical models
Source: Mol Cancer. 2017 Apr 28;16:86. doi: 10.1186/s12943-017-0652-5 (PMC5410089; doi:10.1186/s12943-017-0652-5)
Supplement: Supplementary file 3 — Analysis of differential genomic aberrations in HS-C and LS-C cells. (PDF 255 kb) [file 12943_2017_652_MOESM3_ESM.pdf]

group 1: 2220 probes deleted in HS cells/ unvariated or amplied in LS cells

chr. location

chr 6 q22.1-31

chr 8 p23.1

chr10 q11.21

chr14 q21.1

chr16 q24.3

chr18 q23 q21.2 q21.31 q21.33 q22.1

Term	%	PValue	Genes	Pop Hits	Fold Enrichment
GO:0006643~membrane lipid metabolic process	2,0101	0,05508	ST8SIA6, SMPDL3A, CREM, SERINC1	81	4,616466356
GO:0006893~Golgi to plasma membrane transport	1,005	0,07207	GOPC, OPTN	7	26,70955535
GO:0030212~hyaluronan metabolic process	1,005	0,0917	ITIH5, ITIH2	9	20,7740986
GO:0006629~lipid metabolic process	7,0352	0,09205	CUBN, OLAH, CREM, PDSS1, PHYH, AKR1C3, AKR1C2, AKR1C4, ST8SIA6, SMPDL3A, SERINC1, PIP4K2A, IDI1, AKR1C1	813	1,609800999
GO:0030183~B cell differentiation	1,5075	0,0922	DCLRE1C, KLF6, ITGB1	48	5,842715232
GO:0051329~interphase of mitotic cell cycle	2,0101	0,09683	CUL2, CDC123, ITGB1, NFATC1	103	3,630424998

group 2: 29 probesunvariated or amplified in HS cells/ deleted in LS cells

chr. location

Chr11q25 (unknown associated genes)

group 3: 3200 probes deleted in Hs cells/ unvariated in LS cells

chr. location

chr3 p14.2

chr4 q13.2 q35.2

chr 6 p25.3 p22.3 chr6 p22.2 p22.1 p21.33 p21.32 p21.31 p21.2 p21.1

chr 7 q21.11 q21.12 q31.1

chr 10 p15.3 p15.2 p15.1 p14 p13 p12.33 p12.32 p12.31 p12.2 p 12.1 p11.23 p11.22 p11.21 p11.1

chr 18 q23

chr11 p15.5

chr 12 q21.2 q24.33

chr 13 q11 q12.11 q12.12 q12.13 q12.2 q12.3

chr 14 q31.1

chr 18 q21.32 q23

chr 19 q11 q12 q13.11

chr 22 q12.2

Term	%	PValue	Genes	Pop Hits	Fold Enrichment
GO:0048661~positive regulation of smooth muscle cell proliferation	0,1273	0,00561	FLT1, VEGFA, NOTCH4, FKBPL, AGPAT1	31	6,878471884
GO:0051260~protein homooligomerization	0,2036	0,00683	SCUBE3, ALDH5A1, ALOX5AP, LNX2, PFKP, ACOT13, ITPR3, AKR1C1	95	3,591286373
GO:0002062~chondrocyte differentiation	0,1018	0,00796	FGF9, MAPK14, COL11A2, RUNX2	18	9,477005707
GO:0000082~G1/S transition of mitotic cell cycle	0,1527	0,00977	CCNE1, CUL2, POLE, PIM1, ITGB1, LATS2	56	4,569270609
GO:0030949~positive regulation of vascular endothelial growth factor receptor signaling pathway	0,0764	0,01059	FLT1, FGF9, VEGFA	7	18,27708243
GO:0001569~patterning of blood vessels	0,1018	0,01232	FLT1, NRP1, VEGFA, NOTCH4	21	8,123147749

GO:0008152~metabolic process	5,0904	0,01342	FHIT, CDX2, PGC, HSD17B7P2, MED20, CUL2, EPC1, BRPF3, CUL7, CUL9, MAP3K8, ZNF248, LSM2, ZNF391, B3GALTL, OSBP2, PAN3, C4A, POLH, RPP21, C4B, ZNF507, POLE, MTPAP, PIM1, UBR2, SFRS3, DCLRE1C, RPS18, VEGFA, TMPRSS11F, YME1L1, PFKFB3, OLAH, ZNF76, ASB13, ATP12A, PDSS1, RPL21P28, GAD2, TAKR, MDFI, KLF6, AKR1E2, CFB, YTHDC1, RNF8, RNF6, TPTE2, NOTCH4, FTSJD2, TCF19, PARP4, IDI1, ABCF1, GTF3A, PPARD, SEPHS1, GPLD1, ZEB1, N6AMT2, LATS2, PTER, DIP2C, GPX6, GPX5, USP12, FANCE, MASTL, DHTKD1, C19ORF40, IP6K3, AGPAT1, ZNF33A, PRTFDC1, PEPD, POLR1D, SRPK1, LYZL1, GRM4, C19ORF2, ZSCAN16, AARS2, PRHOXNB, ZMYND11, ETV7, ZBTB9, CREM, ABI1, KIN, MDC1, ALOX5AP, PITRM1, DHX16, MTMR6, UPF2, LRRN3, TRIM27, ZNF25, PPP1R10, MAPK10, PRPF18, PHYH, MSRB2, ZNF165, PSMD13, MAPK14, RPL21, USP49, RBM17, STK38, SLC7A9, MIPEP, MCM10, TAPBP, ZNF187, GATA3, DNAJC1, GUSBL1, MYO3A, NUDT3, NUDT5, CMAH, PNPLA1, TRERF1, ZNF192, ZNF193, MRPS18A, FBXO18, ZNF438, BAT1, PPIL1, PTK7, MRPS10, TFEB, ATHL1, ZNF323, TTBK1, PTPLA, GLO1, STK19, RUNX2, ZMYM2, TAF3, CUBN, TAF8, SACS, TEAD3, FOXP4, PSMB8, SUV39H2, NMT2, ZNF311, ST8SIA6, ZNF318, ADAM22, PIP4K2A, BMI1, HSP90AB1, USPL1, ECHDC3, ZKSCAN3, AKR1C3, AKR1C2, IMMP2L, CRYL1, AKR1CL1, AKR1C4, POU5F1, ITIH5, ITIH2, LTB, AKR1C1, ALDH5A1, PFKP, CDK8, CDC5L, HMGA1, PRKCQ, CAPN11, CHST8, IFT88, TTRAP, CAMK1D, SUPT3H, FKBP5, NFYA, TRDMT1, FKBPL, CEBPA, TNXB, FLT1, ADARB2, FLT3, CEBPG, SIRT3, SLC17A3, SLC17A4, ATP8A2, PSPC1, SCAND3	7647	1,115379251

GO:0016071~mRNA metabolic process	0,4327	0,0138	UPF2, PAN3, ADARB2, PPIL1, CEBPG, MTPAP, YTHDC1, CDC5L, PRPF18, KIN, SFRS3, SRPK1, VEGFA, DHX16, LSM2, RBM17, BAT1	370	1,959434964
GO:0030947~regulation of vascular endothelial growth factor receptor signaling pathway	0,1018	0,01232	FLT1, FGF9, VEGFA	9	14,21550856
GO:0008152~metabolic process	5,0904	0,01342	FHIT, CDX2, STK38, PGC, SLC7A9, MIPEP, MCM10, MED20, EPC1, CUL2, CUL7, BRPF3, ZNF187, CUL9, GATA3, ZNF248, MAP3K8, LSM2, DNAJC1, ZNF391, B3GALTL, GUSBL1, OSBP2, PAN3, C4A, MYO3A, RPP21, POLH, NUDT3, C4B, ZNF507, POLE, NUDT5, MTPAP, PIM1, UBR2, PNPLA1, TRERF1, SFRS3, DCLRE1C, ZNF192, ZNF193, RPS18, MRPS18A, FBXO18, TMPRSS11F, VEGFA, YME1L1, ZNF438, BAT1, PFKFB3, PPIL1, OLAH, ZNF76, PTK7, MRPS10, TFEB, ATHL1, ASB13, ATP12A, PDSS1, RPL21P28, ZNF323, GAD2, TTBK1, PTPLA, GLO1, STK19, RUNX2, MDFI, KLF6, ZMYM2, TAF3, CUBN, CFB, TAF8, SACS, YTHDC1, TEAD3, FOXP4, PSMB8, SUV39H2, NMT2, RNF8, RNF6, ZNF311, ST8SIA6, NOTCH4, TCF19, ZNF318, PARP4, ADAM22, IDI1, PIP4K2A, HSP90AB1, ABCF1, BMI1, GTF3A, PPARD, USPL1, SEPHS1, GPLD1, ZEB1, ZKSCAN3, LATS2, AKR1C3, AKR1C2, IMMP2L, CRYL1, AKR1C4, GPX5, POU5F1, USP12, FANCE, ITIH5, ITIH2, MASTL, DHTKD1, C19ORF40, IP6K3, AKR1C1, AGPAT1, ZNF33A, PRTFDC1, PEPD, POLR1D, ALDH5A1, PFKP, CDK8, CDC5L, HMGA1, SRPK1, PRKCQ, GRM4, CAPN11, CHST8, C19ORF2, ZSCAN16, AARS2, IFT88, PRHOXNB, CAMK1D, TTRAP, SUPT3H, ZMYND11, ETV7, FKBP5, CREM, ZBTB9, ABI1, NFYA, KIN, MDC1, TRDMT1, ALOX5AP, PITRM1, DHX16, MTMR6, FKBPL, CEBPA, UPF2, FLT1, ADARB2, FLT3, CEBPG, LRRN3, TRIM27, PPP1R10, ZNF25, MAPK10, PRPF18, PHYH, MSRB2, ZNF165, SIRT3, PSMD13, MAPK14, RPL21, ATP8A2, PSPC1, USP49, SCAND3, RBM17	7	18,27708243

GO:0044238~primary metabolic process	4,6322	0,01879	ADCY2, UTP18, SLC6A3, ADAMTS16, CTNND2, PRKDC, UBE2V2, UBE2QL1, PRDM16, LPCAT1, RB1CC1, MOS, SDR16C5, SRD5A1, TCEA1, RPS20, TGS1, NSMAF, NSUN2, TERT, TRIP13, PLAG1, LYN, DFFB, PAPD7, ATP6V1H, LYPLA1, LRRC47, SNAI2, MARCH6, MCM4, TP73, PXDNL, MTRR, SPAG9, TMEM68, CCT5, TOM1L1, NME1, PCMTD1, ST18	6923	1,121142232
GO:0051259~protein oligomerization	0,2545	0,02125	SCUBE3, ALDH5A1, ALOX5AP, LNX2, PFKP, TRIM27, ACOT13, ITPR3, PDSS1, AKR1C1	174	2,450949752
GO:0048660~regulation of smooth muscle cell proliferation	0,1273	0,02215	FLT1, VEGFA, NOTCH4, FKBPL, AGPAT1	46	4,635491922
GO:0006986~response to unfolded protein	0,1527	0,02513	BAT5, HSP90AB1, HSPH1, CFB, CSNK2B, CLIC1	71	3,603931748

GO:0043170~macromolecule metabolic process	3,8687	0,02724	FHIT, CDX2, STK38, PGC, MIPEP, MCM10, MED20, EPC1, CUL2, CUL7, BRPF3, ZNF187, CUL9, GATA3, ZNF248, MAP3K8, LSM2, ZNF391, DNAJC1, PAN3, RPP21, POLH, MYO3A, C4A, C4B, ZNF507, POLE, MTPAP, PIM1, UBR2, TRERF1, SFRS3, DCLRE1C, ZNF192, ZNF193, RPS18, MRPS18A, FBXO18, TMPRSS11F, VEGFA, YME1L1, ZNF438, BAT1, ZNF76, PPIL1, PTK7, MRPS10, TFEB, ASB13, RPL21P28, ZNF323, TTBK1, PTPLA, STK19, RUNX2, MDFI, KLF6, ZMYM2, TAF3, CFB, TAF8, SACS, YTHDC1, TEAD3, FOXP4, PSMB8, SUV39H2, NMT2, RNF8, RNF6, ZNF311, ST8SIA6, NOTCH4, TCF19, ZNF318, PARP4, ADAM22, HSP90AB1, ABCF1, BMI1, GTF3A, PPARD, USPL1, SEPHS1, ZEB1, ZKSCAN3, LATS2, IMMP2L, POU5F1, USP12, FANCE, ITIH5, ITIH2, MASTL, LTB, C19ORF40, IP6K3, ZNF33A, PEPD, POLR1D, CDK8, CDC5L, HMGA1, SRPK1, LYZL1, PRKCQ, GRM4, CAPN11, CHST8, C19ORF2, ZSCAN16, IFT88, AARS2, CAMK1D, TTRAP, SUPT3H, ZMYND11, ETV7, FKBP5, CREM, ZBTB9, ABI1, NFYA, KIN, MDC1, TRDMT1, PITRM1, DHX16, MTMR6, FKBPL, CEBPA, UPF2, TNXB, FLT1, ADARB2, FLT3, CEBPG, LRRN3, TRIM27, PPP1R10, ZNF25, MAPK10, PRPF18, MSRB2, ZNF165, SIRT3, PSMD13, MAPK14, RPL21, PSPC1, USP49, SCAND3, RBM17	5710	1,13524902
GO:0051329~interphase of mitotic cell cycle	0,1782	0,03374	CCNE1, CUL2, POLE, CDC123, PIM1, ITGB1, LATS2	103	2,89830757
GO:0006959~humoral immune response	0,1527	0,0375	C4A, CFB, C4B, TFEB, TREM1, PDCD1	79	3,238976634
GO:0051325~interphase	0,1782	0,03802	CCNE1, CUL2, POLE, CDC123, PIM1, ITGB1, LATS2	106	2,816279998

GO:0015959~diadenosine polyphosphate metabolic process	0,0509	0,04621	FHIT, NUDT3	2	42,64652568
GO:0015956~bis(5'-nucleosidyl) oligophosphate metabolic process	0,0509	0,04621	FHIT, NUDT3	2	42,64652568
GO:0042942~D-serine transport	0,0509	0,04621	NFKBIE, SLC7A10	2	42,64652568
GO:0034982~mitochondrial protein processing	0,0509	0,04621	IMMP2L, MIPEP	2	42,64652568
GO:0015961~diadenosine polyphosphate catabolic process	0,0509	0,04621	FHIT, NUDT3	2	42,64652568
GO:0006627~mitochondrial protein processing during import	0,0509	0,04621	IMMP2L, MIPEP	2	42,64652568
GO:0015958~bis(5'-nucleosidyl) oligophosphate catabolic process	0,0509	0,04621	FHIT, NUDT3	2	42,64652568
GO:0051726~regulation of cell cycle	0,3563	0,04775	GTPBP4, GMNN, PIM1, ITGB1, LATS2, PRKCQ, MAD2L1BP, CCND3, CUL7, MDC1, MAPK14, CUL9, CDC123, LTB	331	1,803780542
GO:0046942~carboxylic acid transport	0,2036	0,0573	PPARD, AKR1C4, SLC26A8, NFKBIE, SLC7A1, SLC7A9, SLC7A10, AKR1C1	147	2,320899357
GO:0015849~organic acid transport	0,2036	0,059	PPARD, AKR1C4, SLC26A8, NFKBIE, SLC7A1, SLC7A9, SLC7A10, AKR1C1	148	2,305217604

GO:0051262~protein tetramerization	0,1018	0,06238	ALDH5A1, PFKP, ACOT13, PDSS1	39	4,374002634
GO:0008284~positive regulation of cell proliferation	0,4072	0,06368	BMI1, PPARD, NRP1, CDX2, FLT1, IL2RA, FLT3, FGF9, ITGB1, PRKCQ, VEGFA, NOTCH4, CDC123, RUNX2, FKBPL, AGPAT1	414	1,648174905
GO:0010464~regulation of mesenchymal cell proliferation	0,0764	0,06518	FGF9, VEGFA, ZEB1	18	7,10775428
GO:0007049~cell cycle	0,6617	0,06582	ZMYND11, KIFC1, PARD3, AIF1, GMNN, POLE, PIM1, UBR2, CDC5L, ITGB1, CENPJ, LATS2, PSMB8, SUV39H2, RNF8, CCNE1, CUL2, PSMD13, CCND3, SLC26A8, MDC1, MAP3K8, CDC123, SKA3, ZNF318, PARD6G	776	1,428878438
GO:0006468~protein amino acid phosphorylation	0,5854	0,06679	MDFI, PAN3, FLT1, STK38, MYO3A, FLT3, LRRN3, PIM1, PTK7, CDK8, ABI1, MAPK10, LATS2, SRPK1, GRM4, PRKCQ, TTBK1, MAPK14, MAP3K8, MASTL, STK19, IP6K3, CAMK1D	667	1,470569851

GO:0044260~cellular macromolecule metabolic process	3,4614	0,07348	FHIT, CDX2, STK38, MIPEP, MCM10, MED20, EPC1, CUL2, CUL7, BRPF3, ZNF187, CUL9, GATA3, ZNF248, MAP3K8, LSM2, ZNF391, DNAJC1, PAN3, RPP21, POLH, MYO3A, ZNF507, POLE, MTPAP, PIM1, UBR2, TRERF1, SFRS3, DCLRE1C, ZNF192, ZNF193, RPS18, MRPS18A, FBXO18, VEGFA, ZNF438, BAT1, ZNF76, PPIL1, PTK7, TFEB, MRPS10, ASB13, RPL21P28, ZNF323, TTBK1, PTPLA, STK19, RUNX2, MDFI, KLF6, ZMYM2, TAF3, TAF8, SACS, YTHDC1, TEAD3, FOXP4, PSMB8, SUV39H2, NMT2, RNF8, RNF6, ZNF311, ST8SIA6, NOTCH4, TCF19, ZNF318, PARP4, HSP90AB1, BMI1, ABCF1, GTF3A, PPARD, USPL1, SEPHS1, ZEB1, ZKSCAN3, LATS2, IMMP2L, POU5F1, USP12, FANCE, MASTL, C19ORF40, IP6K3, ZNF33A, POLR1D, CDK8, CDC5L, HMGA1, SRPK1, GRM4, PRKCQ, CHST8, C19ORF2, ZSCAN16, AARS2, TTRAP, CAMK1D, SUPT3H, ZMYND11, ETV7, FKBP5, CREM, ZBTB9, ABI1, NFYA, KIN, MDC1, TRDMT1, DHX16, MTMR6, FKBPL, CEBPA, UPF2, FLT1, ADARB2, FLT3, CEBPG, LRRN3, TRIM27, ZNF25, PPP1R10, MAPK10, PRPF18, MSRB2, SIRT3, ZNF165, PSMD13, MAPK14, RPL21, PSPC1, USP49, SCAND3, RBM17	5214	1,112375814
GO:0006397~mRNA processing	0,3309	0,07454	ADARB2, PPIL1, MTPAP, DHX16, YTHDC1, CDC5L, LSM2, PRPF18, KIN, SRPK1, SFRS3, RBM17, BAT1	321	1,727117862

GO:0044237~cellular metabolic process	4,3013	0,08389	FHIT, CDX2, STK38, SLC7A9, MIPEP, MCM10, MED20, TAPBP, EPC1, CUL2, CUL7, BRPF3, ZNF187, CUL9, GATA3, ZNF248, MAP3K8, LSM2, DNAJC1, ZNF391, B3GALTL, PAN3, RPP21, POLH, MYO3A, NUDT3, ZNF507, POLE, NUDT5, MTPAP, PIM1, UBR2, TRERF1, SFRS3, DCLRE1C, ZNF192, ZNF193, RPS18, MRPS18A, FBXO18, VEGFA, ZNF438, BAT1, PFKFB3, ZNF76, OLAH, PPIL1, PTK7, MRPS10, TFEB, ASB13, ATP12A, PDSS1, RPL21P28, ZNF323, GAD2, TTBK1, PTPLA, GLO1, STK19, RUNX2, MDFI, KLF6, ZMYM2, TAF3, TAF8, SACS, YTHDC1, TEAD3, FOXP4, PSMB8, SUV39H2, NMT2, RNF8, RNF6, ZNF311, TPTE2, ST8SIA6, NOTCH4, FTSJD2, TCF19, ZNF318, PARP4, IDI1, PIP4K2A, HSP90AB1, ABCF1, BMI1, GTF3A, PPARD, USPL1, SEPHS1, GPLD1, ZEB1, N6AMT2, ZKSCAN3, LATS2, AKR1C3, AKR1C2, IMMP2L, CRYL1, AKR1C4, POU5F1, USP12, FANCE, MASTL, DHTKD1, C19ORF40, IP6K3, AKR1C1, AGPAT1, ZNF33A, PRTFDC1, PEPD, POLR1D, ALDH5A1, PFKP, CDK8, CDC5L, HMGA1, SRPK1, PRKCQ, GRM4, CHST8, C19ORF2, ZSCAN16, AARS2, PRHOXNB, CAMK1D, TTRAP, SUPT3H, ZMYND11, ETV7, FKBP5, CREM, ZBTB9, ABI1, NFYA, KIN, MDC1, TRDMT1, ALOX5AP, DHX16, MTMR6, FKBPL, CEBPA, UPF2, FLT1, ADARB2, FLT3, CEBPG, LRRN3, TRIM27, PPP1R10, ZNF25, MAPK10, PRPF18, MSRB2, ZNF165, SIRT3, PSMD13, SLC17A3, SLC17A4, MAPK14, RPL21, ATP8A2, PSPC1, USP49, SCAND3, RBM17	6636	1,086085419
GO:0046130~purine ribonucleoside catabolic process	0,0509	0,09029	FHIT, NUDT3	4	21,32326284
GO:0032329~serine transport	0,0509	0,09029	NFKBIE, SLC7A10	4	21,32326284
GO:0006152~purine nucleoside catabolic process	0,0509	0,09029	FHIT, NUDT3	4	21,32326284

GO:0006282~regulation of DNA repair	0,0764	0,09265	RNF8, POLH, CEBPG	22	5,81543532
GO:0006631~fatty acid metabolic process	0,2291	0,09341	AKR1C3, AKR1C2, CRYL1, PPARD, ALDH5A1, MAPK14, OLAH, CREM, ALOX5AP	198	1,93847844
GO:0045893~positive regulation of transcription, DNA-dependent	0,4327	0,09485	CEBPA, KLF6, CDX2, TAF8, TFEB, TEAD3, NFYA, ZEB1, HMGA1, TRERF1, CCNE1, EPC1, RNF6, MAPK14, VEGFA, NOTCH4, RUNX2	477	1,519897142
GO:0051216~cartilage development	0,1273	0,09489	FGF9, MAPK14, ZEB1, COL11A2, RUNX2	74	2,881522005

group 4: 1324 probes unvariated in HS cells/ amplified in LS cells

Chr 1 p36.32

Chr 5 p15.33 p15.32 p15.31 p15.2 p15.1

Chr 8 q11.1 q11.21 q11.22 q11.23 q12.1

Chr 11 q11

Chr 16 p11.2 p11.1

Chr 17 q21.33 q22

Term	%	PValue	Genes	Pop Hits	Fold Enrichment
GO:0051716~cellular response to stimulus	1,3854	0,00194	RP1, ADCY2, LYN, RB1CC1, PAPD7, PRKDC, UBE2V2, STXBP4, TP73, PXDNL, TRIP13	820	3,15601626

GO:0006302~double-strand break repair	0,5038	0,0022	PAPD7, PRKDC, UBE2V2, TRIP13	62	15,17849462
GO:0006259~DNA metabolic process	1,0076	0,00496	DFFB, PAPD7, PRKDC, UBE2V2, MCM4, TERT, TP73, TRIP13	506	3,719631094
GO:0033554~cellular response to stress	1,0076	0,009	LYN, RB1CC1, PAPD7, PRKDC, UBE2V2, TP73, PXDNL, TRIP13	566	3,32532391
GO:0044237~cellular metabolic process	4,7859	0,01091	ADCY2, UTP18, SLC6A3, CTNND2, PRKDC, UBE2V2, UBE2QL1, PRDM16, LPCAT1, RB1CC1, MOS, SRD5A1, TCEA1, RPS20, TGS1, NSMAF, NSUN2, TERT, TRIP13, PLAG1, LYN, DFFB, PAPD7, ATP6V1H, LYPLA1, LRRC47, SNAI2, MARCH6, MCM4, TP73, PXDNL, MTRR, SPAG9, CCT5, TOM1L1, NME1, PCMTD1, ST18	6636	1,347217199
GO:0042493~response to drug	0,6297	0,01259	LYN, NME1, SLC6A3, PAPD7, SRD5A1	216	5,445987654
GO:0006974~response to DNA damage stimulus	0,7557	0,01957	LYN, PAPD7, PRKDC, UBE2V2, TP73, TRIP13	373	3,784450402
GO:0008152~metabolic process	5,1637	0,02313	ADCY2, UTP18, SLC6A3, ADAMTS16, CTNND2, PRKDC, UBE2V2, UBE2QL1, PRDM16, LPCAT1, RB1CC1, MOS, SDR16C5, SRD5A1, TCEA1, RPS20, TGS1, NSMAF, NSUN2, TERT, TRIP13, PLAG1, LYN, DFFB, PAPD7, ATP6V1H, LYPLA1, LRRC47, SNAI2, MARCH6, MCM4, TP73, PXDNL, MTRR, SPAG9, TMEM68, CCT5, TOM1L1, NME1, PCMTD1, ST18	7647	1,261400985

GO:0044238~primary metabolic process	4,7859	0,02387	ADCY2, UTP18, SLC6A3, ADAMTS16, CTNND2, PRKDC, UBE2V2, UBE2QL1, PRDM16, LPCAT1, RB1CC1, MOS, SRD5A1, TCEA1, RPS20, TGS1, NSMAF, NSUN2, TERT, TRIP13, PLAG1, LYN, DFFB, PAPD7, ATP6V1H, LYPLA1, LRRC47, SNAI2, MARCH6, MCM4, TP73, MTRR, SPAG9, CCT5, TOM1L1, NME1, PCMTD1, ST18	6923	1,291366941
GO:0006281~DNA repair	0,6297	0,03077	PAPD7, PRKDC, UBE2V2, TP73, TRIP13	284	4,142018779
GO:0044249~cellular biosynthetic process	2,7708	0,03535	PLAG1, ADCY2, SLC6A3, CTNND2, PAPD7, ATP6V1H, LRRC47, SNAI2, PRDM16, MCM4, TP73, MTRR, LPCAT1, NME1, RB1CC1, SRD5A1, TCEA1, RPS20, ST18, TGS1, TERT, TRIP13	3442	1,503738137
GO:0034641~cellular nitrogen compound metabolic process	2,8967	0,03688	PLAG1, ADCY2, UTP18, DFFB, SLC6A3, CTNND2, PAPD7, PRKDC, ATP6V1H, UBE2V2, SNAI2, PRDM16, MCM4, TP73, MTRR, NME1, RB1CC1, TCEA1, ST18, TGS1, NSUN2, TERT, TRIP13	3670	1,474423252
GO:0051171~regulation of nitrogen compound metabolic process	2,3929	0,03804	PLAG1, ADCY2, OPRK1, SLC6A3, IRX2, IRX1, CTNND2, PRKDC, UBE2V2, PRDM16, SNAI2, MCM4, TP73, MBTD1, NME1, RB1CC1, TCEA1, ST18, TGS1	2839	1,574521545
GO:0001510~RNA methylation	0,2519	0,04103	TGS1, NSUN2	10	47,05333333
GO:0080090~regulation of primary metabolic process	2,6448	0,04217	PLAG1, ADCY2, LYN, OPRK1, SLC6A3, IRX2, IRX1, CTNND2, PRKDC, UBE2V2, UBE2QL1, PRDM16, SNAI2, MCM4, TP73, MBTD1, NME1, RB1CC1, TCEA1, ST18, TGS1	3291	1,501245822
GO:0044271~nitrogen compound biosynthetic process	0,6297	0,04672	ADCY2, NME1, SLC6A3, ATP6V1H, MTRR	325	3,619487179

GO:0009058~biosynthetic process	2,7708	0,04693	PLAG1, ADCY2, SLC6A3, CTNND2, PAPD7, ATP6V1H, LRRC47, SNAI2, PRDM16, MCM4, TP73, MTRR, LPCAT1, NME1, RB1CC1, SRD5A1, TCEA1, RPS20, ST18, TGS1, TERT, TRIP13	3542	1,461283644
GO:0006807~nitrogen compound metabolic process	2,8967	0,04957	PLAG1, ADCY2, UTP18, DFFB, SLC6A3, CTNND2, PAPD7, PRKDC, ATP6V1H, UBE2V2, SNAI2, PRDM16, MCM4, TP73, MTRR, NME1, RB1CC1, TCEA1, ST18, TGS1, NSUN2, TERT, TRIP13	3778	1,432274572
GO:0019222~regulation of metabolic process	2,7708	0,05793	PLAG1, ADCY2, LYN, OPRK1, SLC6A3, IRX2, IRX1, CTNND2, PRKDC, UBE2V2, UBE2QL1, PRDM16, SNAI2, MCM4, TP73, SPAG9, MBTD1, NME1, RB1CC1, TCEA1, ST18, TGS1	3621	1,429402559
GO:0006139~nucleobase, nucleoside, nucleotide and nucleic acid metabolic process	2,6448	0,05844	PLAG1, ADCY2, UTP18, DFFB, CTNND2, PAPD7, PRKDC, ATP6V1H, UBE2V2, SNAI2, PRDM16, MCM4, TP73, NME1, RB1CC1, TCEA1, ST18, TGS1, NSUN2, TERT, TRIP13	3409	1,449281314
GO:0044260~cellular macromolecule metabolic process	3,6524	0,06297	UTP18, CTNND2, PRKDC, UBE2V2, UBE2QL1, PRDM16, RB1CC1, MOS, TCEA1, RPS20, TGS1, NSUN2, TERT, TRIP13, PLAG1, LYN, DFFB, PAPD7, LRRC47, MARCH6, SNAI2, MCM4, TP73, SPAG9, CCT5, TOM1L1, NME1, PCMTD1, ST18	5214	1,308541107
GO:0019219~regulation of nucleobase, nucleoside, nucleotide and nucleic acid metabolic process	2,267	0,06535	PLAG1, ADCY2, IRX2, OPRK1, IRX1, CTNND2, PRKDC, UBE2V2, PRDM16, SNAI2, MCM4, TP73, MBTD1, NME1, RB1CC1, TCEA1, ST18, TGS1	2814	1,504904051
GO:0032868~response to insulin stimulus	0,3778	0,06552	LYN, PRKDC, STXBP4	100	7,058

GO:0031323~regulation of cellular metabolic process	2,6448	0,06742	PLAG1, ADCY2, LYN, OPRK1, SLC6A3, IRX2, IRX1, CTNND2, PRKDC, UBE2V2, PRDM16, SNAI2, MCM4, TP73, SPAG9, MBTD1, NME1, RB1CC1, TCEA1, ST18, TGS1	3464	1,426270208
GO:0002762~negative regulation of myeloid leukocyte differentiation	0,2519	0,06876	NME1, PRDM16	17	27,67843137
GO:0080135~regulation of cellular response to stress	0,3778	0,06899	LYN, UBE2V2, TP73	103	6,852427184
GO:0042221~response to chemical stimulus	1,2594	0,08323	ADCY2, LYN, NME1, SLC6A3, PAPD7, PRKDC, SRD5A1, STXBP4, SLC6A19, PXDNL	1281	1,836586001
GO:0015837~amine transport	0,3778	0,08718	LYN, SLC6A3, SLC6A19	118	5,981355932
GO:0022414~reproductive process	0,8816	0,09751	SPAG9, NME1, SLC6A3, OPRK1, PRKDC, SRD5A1, TRIP13	762	2,161242345
GO:0030261~chromosome condensation	0,2519	0,0995	DFFB, PAPD7	25	18,82133333
GO:0000003~reproduction	0,8816	0,09984	SPAG9, NME1, SLC6A3, OPRK1, PRKDC, SRD5A1, TRIP13	767	2,147153412
